# Vedolizumab and ART in recent HIV-1 infection unveil the role of **α**4**β**7 in reservoir size

**DOI:** 10.1172/jci.insight.182312

**Published:** 2024-07-09

**Authors:** Maria Reyes Jimenez-Leon, Carmen Gasca-Capote, Cristina Roca-Oporto, Nuria Espinosa, Salvador Sobrino, Maria Fontillon-Alberdi, Ce Gao, Isabelle Roseto, Gregory Gladkov, Inmaculada Rivas-Jeremias, Karin Neukam, Jose German Sanchez-Hernandez, Raul Rigo-Bonnin, Antonio J. Cervera-Barajas, Rosario Mesones, Federico García, Ana Isabel Alvarez-Rios, Sara Bachiller, Joana Vitalle, Alberto Perez-Gomez, María Inés Camacho-Sojo, Isabel Gallego, Christian Brander, Ian McGowan, Beatriz Mothe, Pompeyo Viciana, Xu Yu, Mathias Lichterfeld, Luis F. Lopez-Cortes, Ezequiel Ruiz-Mateos

**Affiliations:** 1 Institute of Biomedicine of Seville, Virgen del Rocío University Hospital, Consejo Superior de Investigaciones Científicas, University of Seville, Clinical Unit of Infectious Diseases, Microbiology and Parasitology, Seville, Spain.; 2 Digestive Service and; 3 Service of Pathological Anatomy, Virgen del Rocío University Hospital, Seville, Spain.; 4 Ragon Institute of MGH, MIT and Harvard, Cambridge, Massachusetts, USA.; 5 Infectious Disease Division, Brigham and Women’s Hospital, Boston, Massachusetts, USA.; 6 Pharmacy service, University Hospital of Salamanca-Institute for Biomedical Research of Salamanca, Salamanca, Spain.; 7 Department of Clinical Laboratory, Hospital Universitari de Bellvitge, Instituto de Investigación Biomédica de Bellvitge, Universitat de Barcelona, Barcelona, Spain.; 8 Clinical Trials Units, Virgen del Rocío University Hospital, Seville, Spain.; 9 Departament of Microbiology, San Cecilio University Hospital, Instituto de Investigación Ibs, Granada, Ciber de Enfermedades Infecciosas, Centro de Investigación Biomédica en Red de Enfermedades Infecciosas, Granada, Spain.; 10 Biochemistry Service, Virgen del Rocío University Hospital, Seville, Spain.; 11 AELIX Therapeutics S.L., Barcelona, Spain.; 12 Infectious Diseases Department and IrsiCaixa AIDS Research Institute, Hospital Germans Trias I Pujol, Badalona, Spain.; 13 Centro de Investigación Biomédica en Red de Enfermedades Infecciosas, Spain.

**Keywords:** AIDS/HIV, Immunology, Immunotherapy

## Abstract

**BACKGROUND:**

We evaluated the safety and viral rebound, after analytical treatment interruption (ATI), of vedolizumab and ART in recent HIV-1 infection. We used this model to analyze the effect of α4β7 on the HIV-1 reservoir size.

**METHODS:**

Participants started ART with monthly vedolizumab infusions, and ATI was performed at week 24. Biopsies were obtained from ileum and cecum at baseline and week 24. Vedolizumab levels, HIV-1 reservoir, flow cytometry, and cell-sorting and antibody competition experiments were assayed.

**RESULTS:**

Vedolizumab was safe and well tolerated. No participant achieved undetectable viremia off ART 24 weeks after ATI. Only a modest effect on the time to achieve more than 1,000 HIV-1 RNA copies/mL and the proportion of participants off ART was observed, being higher in the vedolizumab group compared with historical controls. Just before ATI, α4β7 expression was associated with HIV-1 DNA and RNA in peripheral blood and with PD1 and TIGIT levels. Importantly, a complete blocking of α4β7 was observed on peripheral CD4^+^ T cells but not in gut (ileum and cecum), where α4β7 blockade and vedolizumab levels were inversely associated with HIV-1 DNA.

**CONCLUSION:**

Our findings support α4β7 as an important determinant in HIV-1 reservoir size, suggesting the complete α4β7 blockade in tissue as a promising tool for HIV-cure combination strategies.

**TRIAL REGISTRATION:**

ClinicalTrials.gov NCT03577782.

**FUNDING:**

This work was supported by the Instituto de Salud Carlos III (Fondo Europeo de Desarrollo Regional, “a way to make Europe,” research contracts FI17/00186 and FI19/00083 and research projects PI18/01532, PI19/01127, PI22/01796), Conserjería de Economía, Conocimiento, Empresas y Universidad, Junta de Andalucía (research projects P20/00906), the Red Temática de Investigación Cooperativa en SIDA (RD16/0025/0020), and the Spanish National Research Council.

## Abstract 2

The blockade of α4β7 by vedolizumab reveal the important role of α4β7 in HIV-1 reservoir size in peripheral blood and tissue.

## Introduction

Antiretroviral therapy (ART) suppresses HIV-1 replication to undetectable plasma levels but fails to eradicate the virus (1). HIV-1 remains transcriptionally active, primarily from defective HIV proviruses (2), or latent in anatomical and cellular reservoirs (3, 4). However, HIV rebounds after ART interruption in most people living with HIV (PLWH) (5, 6). Therapeutic strategies are being explored to achieve HIV eradication or permanent viral remission in the absence of ART, as occurs in persistent HIV-1 controllers (7). HIV-1 preferentially infects activated memory CD4^+^ T cells, which are enriched in gastrointestinal tissues (GITs) (8, 9). One of the pathways used by CD4^+^ T cells for trafficking into GITs is the interaction between α4β7 integrin, expressed on CD4^+^ T cells, and the mucosal vascular addressin cell adhesion molecule 1 (MAdCAM-1), expressed primarily on high endothelial venules within GITs (10). Additionally, α4β7 integrin is also incorporated in HIV-1 virions (11). HIV-1 gp120 can bind to α4β7 integrin, expressed on CD4^+^ T cells, leading to a rapid activation of lymphocyte function-associated antigen 1 (LFA-1), the integrin involved in the establishment of virological “synapses” and promoting cell-to-cell transmission of infection (12). These are key aspects in HIV-1 immunopathogenesis that need to be tackled to achieve a sustained virological remission since a high number of the target cells for HIV-1 infection are in the GITs. In this sense, CD4^+^α4β7^+^ T cells were found to harbor 3 times more SIV DNA than α4β7^–^ T cell subsets (13). Besides, it has been shown that high levels of CD4^+^α4β7^+^ T cells increased the susceptibility to HIV-1 infection in nonhuman primates and heterosexual women (14–16). In addition, treatment with α4β7 blocking molecules significantly reduced SIV-DNA levels in the gut (13, 17–19). However, the effect of blocking α4β7 expression on the HIV-1 reservoir landscape in peripheral blood and tissue in humans remains uncertain. These findings led to the hypothesis that α4β7 could be targeted to achieve a permanent virological remission off ART in humans. Vedolizumab is a humanized monoclonal antibody against α4β7 that is licensed for the treatment of inflammatory bowel disease (20–22). The therapeutic role of an α4β7 monoclonal antibody in HIV cure research remains unclear. In a recent clinical trial, no sustained viral remission was found after ART and vedolizumab treatment in ART-suppressed participants with chronic HIV infection (23), and the seminal efficacy data generated in a nonhuman primate model (19) could not be reproduced (24–26). In the present study, we evaluated the safety and efficacy in viral rebound, after analytical treatment interruption (ATI), of vedolizumab combined with ART on recently infected PLWH. None of the participants achieved undetectable viremia off ART at the end of the follow-up. However, importantly, α4β7 expression was associated with DNA and RNA HIV-1 levels in peripheral blood and in 2 gut locations (ileum and cecum). In addition, α4β7 levels were associated with PD1 and TIGIT protein levels, immune checkpoints molecules previously associated with the HIV-1 reservoir (27). Finally, just before ATI, despite the complete α4β7 blockade on peripheral CD4^+^ T cells, α4β7 was not entirely blocked in the gut, where the percentage of α4β7 blockade and vedolizumab levels were inversely associated with HIV-1 DNA levels. Therefore, using this model we describe key insights into the role of α4β7 in vivo in the human HIV-1 reservoir.

## Results

### Participants’ characteristics and safety of vedolizumab and ART in PLWH.

**Figure 1:**
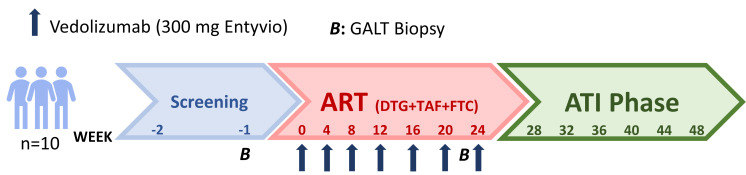
Clinical trial design. Ten individuals with HIV-1 diagnosis in acute/recent infection phase were enrolled. Participants started ART together with vedolizumab infusions (300 mg) at week 0, 4, 8, 12, 16, 20, and 24. At week 24, ART and vedolizumab treatments were interrupted. Biopsies were obtained from ileum and cecum at week 0 and 24. GALT, gut-associated lymphoid tissue; ART, antiretroviral therapy; ATI, analytic treatment interruption.

Ten PLWH naive for ART (9 cisgender male participants and 1 cisgender female participant) were enrolled between September 2018 and June 2019 (Figure 1); all participants completed the study follow-up period. A total of 7 monthly vedolizumab infusions were administrated, in addition to ART, to each participant for 24 weeks. No adverse effects were observed during the infusions or postinfusion periods (Supplemental Table 1; supplemental material available online with this article; https://doi.org/10.1172/jci.insight.182312DS1). Furthermore, no participant had detectable anti-vedolizumab antibodies at baseline (BL) or throughout follow-up (data not shown). Date of HIV-1 infection was estimated as the average between a HIV-1^–^ and HIV-1^+^ serologic test (maximum time frame of 6 months) and/or 15 days before onset of symptoms compatible with acute retroviral syndrome. The median time from HIV-1 infection to study initiation was 75 days (IQR 40–82 days). Demographic, immunological, and virological characteristics of the study participants (vedolizumab group) are summarized in Table 1. In summary, vedolizumab was safe and well tolerated in people that started ART and vedolizumab after recent HIV infection diagnosis.

### Efficacy after the ATI.

**Figure 2:**
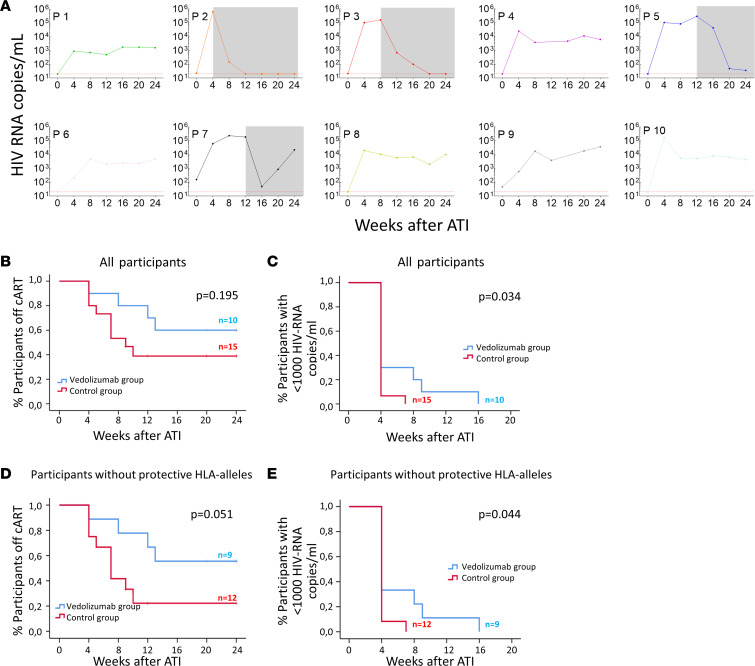
Plasma viral load, proportion of participants off ART, and time to viral load rebound after ATI. (**A**) Longitudinal plasma viremia evolution after ATI. Four participants restarted ART (gray area) because of an increase of viral load (>10^5^ HIV-1 RNA copies/mL). The horizontal red line indicates the limit of detection (20 HIV-1 RNA copies/mL). (**B**) Kaplan-Meier analysis of the proportion of participants off ART after ATI compared with the historical control group. (**C**) Kaplan-Meier analysis between the vedolizumab and historical control groups of the time to first viral load of more than 1,000 HIV-1 RNA copies/mL. (**D** and **E**) Kaplan-Meier analysis considering only participants without protective alleles (HLA-B*27 and HLA-B*57). In this analysis, participants 36, 16, and 17 from the historical control cohort and participant 4 from the vedolizumab group were excluded. Wilcoxon’s test, log-rank, and Kaplan-Meier curves were used to assess differences during the follow-up period. BL, baseline; W, week; ATI, analytic treatment interruption.

ART and vedolizumab were interrupted at week 24, and participants were followed every 4 weeks during the ATI period for up to 24 weeks. The plasma viral load (pVL) kinetics before ATI is shown in Supplemental Figure 1A. ART was restarted when pVL was more than 100,000 HIV-1 RNA copies/mL in 2 consecutive measurements 1 month apart. All participants had detectable viremia during the ATI, and none achieved undetectable viremia (<20 HIV-1 RNA copies/mL) after 24 weeks of follow-up in the absence of treatment (Figure 2A). Four participants resumed ART due to the virological criteria, and the other 6 participants completed the follow-up with pVL of 1,590 HIV-1 RNA copies/mL (participant 1 [P1]); 6,250 HIV-1 RNA copies/mL (P4); 4,670 HIV-1 RNA copies/mL (P6); 10,000 HIV-1 RNA copies/mL (P8); 36,450 HIV-1 RNA copies/mL (P9); and 4,300 HIV-1 RNA copies/mL (P10) at week 48, respectively (Figure 2A). Participant number 7 restarted treatment at week 36 (12 weeks after ATI) and showed new viral recrudesce at week 40, compatible with self-reported intermittent low adherence to the treatment during the whole clinical trial. For that reason, P7 was removed from HIV-1 reservoir analysis. No ART resistance mutations were detected at BL, week 24, and week 48 in this participant (data not shown). Overall, there were no decreases in CD4^+^ T cell counts at week 24, 28, and 48 compared with the BL; in fact, we observed a significant increase in CD4^+^ T cell counts at week 24 and 28 (Supplemental Figure 1B). We did not observe a significant decrease from ATI to week 48 (Supplemental Figure 1B). Therefore, in this study we did not observe sustained viral remission during ATI after 24 weeks of ART and vedolizumab treatment in recently infected PLWH.

Subsequently, in a post hoc analysis, we compared the pVL kinetics during the ATI from the vedolizumab group with historical controls from the placebo arm of the AELIX-002 study (NCT03204617), which also included a 24-week ATI (28). Both groups were matched by estimated time since HIV-1 acquisition at the moment of starting ART, sex, and age (Table 1). At the moment of ATI, CD4^+^ T cell counts and the CD4/CD8 ratio were higher in the historical controls, as these participants had been ART suppressed for 1 year more than the vedolizumab group (Table 1). For the purpose of this post hoc comparison, time off ART was analyzed with the same virological ART resumption criteria as the vedolizumab group. We did not observe significant differences in the proportion of participants remaining off ART between the 2 studies (Figure 2B). However, longer time to first pVL of more than 1,000 HIV-1 RNA copies/mL was observed in the vedolizumab group (*P* = 0.034) (Figure 2C). Time off ART was 24 weeks (IQR, 8–24 weeks) and 8 weeks (IQR, 5–20 weeks) in our study and the historical control cohort, respectively (*P* = 0.06; Supplemental Table 2). A nonsignificant increase in the time to reach more than 2,000 HIV-1 RNA copies/mL (*P* = 0.074) was observed in the vedolizumab group, and no differences were observed in the time to first pVL of more than 10,000 or 20,000 HIV-1 RNA copies/mL (*P* = 0.333 and *P* = 0.303, respectively) (Supplemental Figure 1, C–E) and other parameters (Supplemental Table 2).

HLA protective alleles have been associated with the spontaneous control of HIV viremia (29, 30). Individuals with these alleles may bias viral rebound kinetics after ATI. Considering only participants without protective HLA alleles (P36, P16, and P17 from the historical control cohort and P4 from our study were excluded), with the aim of avoiding confusing factors that could favor the viremia control, the differences between pVL kinetics increased between groups. There was a higher but not significant (*P* = 0.051) proportion of participants remaining off ART in the vedolizumab group (Figure 2D). Interestingly, the time off ART (median [IQR], 24 weeks [8–24 weeks] vs. 7 weeks [4–10 weeks]) and to peak pVL (median [IQR], 8 weeks [4–14 weeks] vs. 4 weeks [4–7 weeks]) were higher in the vedolizumab group compared with the historical controls (*P* = 0.027 and *P* = 0.047), respectively, the same as the time to first pVL of more than 1,000 HIV-1 RNA copies/mL (*P* = 0.044) (Figure 2E). A nonsignificant increase was observed in the time to reach more than 2,000 HIV-1 RNA copies/mL (*P* = 0.094) in the vedolizumab group, and no differences were observed in the time to first pVL of more than 10,000 or 20,000 HIV-1 RNA copies/mL (*P* = 0.263 and *P* = 0.285, respectively). It is important to note, that pVL before ART, in these participants without protective HLA alleles, was higher in the vedolizumab group compared with the historical control group (median [IQR], 6.09 HIV-1 RNA copies/mL [5.12–6.90 HIV-1 RNA copies/mL] vs. 4.95 HIV-1 RNA copies/mL [4.43–5.73 HIV-1 RNA copies/mL], *P* = 0.030).

### Combined therapy resulted in decreased HIV-1 reservoir levels.

**Figure 3:**
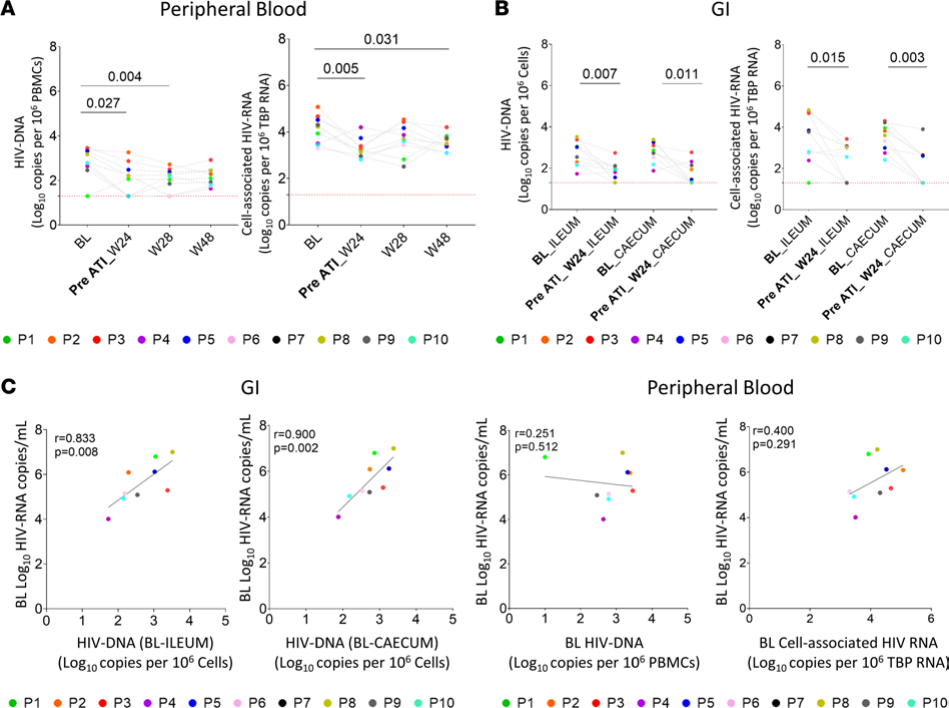
Dynamics of HIV-1 reservoir. (**A**) Total cell-associated HIV-1 DNA and RNA in PBMCs during the follow-up period. (**B**) Cell-associated HIV-1 DNA and RNA in ileum and cecum cells at BL and week 24. (**C**) Associations between HIV-1 reservoir in GITs (cell-associated HIV-1 DNA) and PBMCs (cell-associated HIV-1 DNA and RNA) and plasma viral load at BL. Horizontal red line indicates the limit of detection. Friedman’s test with Dunn’s multiple comparisons test correction was used to assess differences during the follow-up period and Mann-Whitney *U* test was used between GIT locations. *P* values are shown within the graphs. BL, baseline; W, week; ATI, analytic treatment interruption.

Next, although no sustained viral remission was found, we took advantage of the study design to explore the relationship between immunological factors in the intervention cohort, focusing on α4β7^+^ expression, and HIV-1 reservoir levels in peripheral blood and GIT. Regarding HIV reservoir levels, a decrease in total HIV-1 DNA was observed in PBMCs at weeks 24 and 28. (Figure 3A, left). A similar pattern was observed in cell-associated HIV-1 RNA except for week 28 (Figure 3A, right). This may be due to the fact that all participants at week 28 were without ART but with detectable pVL (Figure 2A). Interestingly, in all of the studied time points, participants who restarted ART showed higher levels of total HIV-1 DNA in PBMCs than participants who reached study week 48 of follow-up without ART (Supplemental Figure 2A, left). The same kinetic was observed for cell-associated HIV-1 RNA but only for BL and week 28 (Supplemental Figure 2A, right). HIV-1 reservoir was also assayed in ileum and cecum cells (Figure 3B). A significant decrease was observed in both locations in total HIV-1 DNA and cell-associated HIV-1 RNA at week 24 respect to BL (Figure 3B, left and right, respectively). We did not observe differences in HIV-1 reservoir levels (DNA or RNA) between ileum and cecum, neither at BL nor week 24. Participants who restarted ART presented similar levels of HIV-1 reservoir in GIT (DNA or RNA) as those who did not restart ART, with no significant differences at BL and week 24 in ileum or cecum (Supplemental Figure 2B, left and right panel, respectively). There was a strong positive correlation between total HIV-1 DNA reservoir in ileum and cecum and the pVL at BL (Figure 3C, left). Interestingly, this correlation was not observed with the HIV-1 reservoir (DNA or RNA) in peripheral blood (Figure 3C, right).

### Effect of combined therapy on β7 integrin expression.

**Figure 4:**
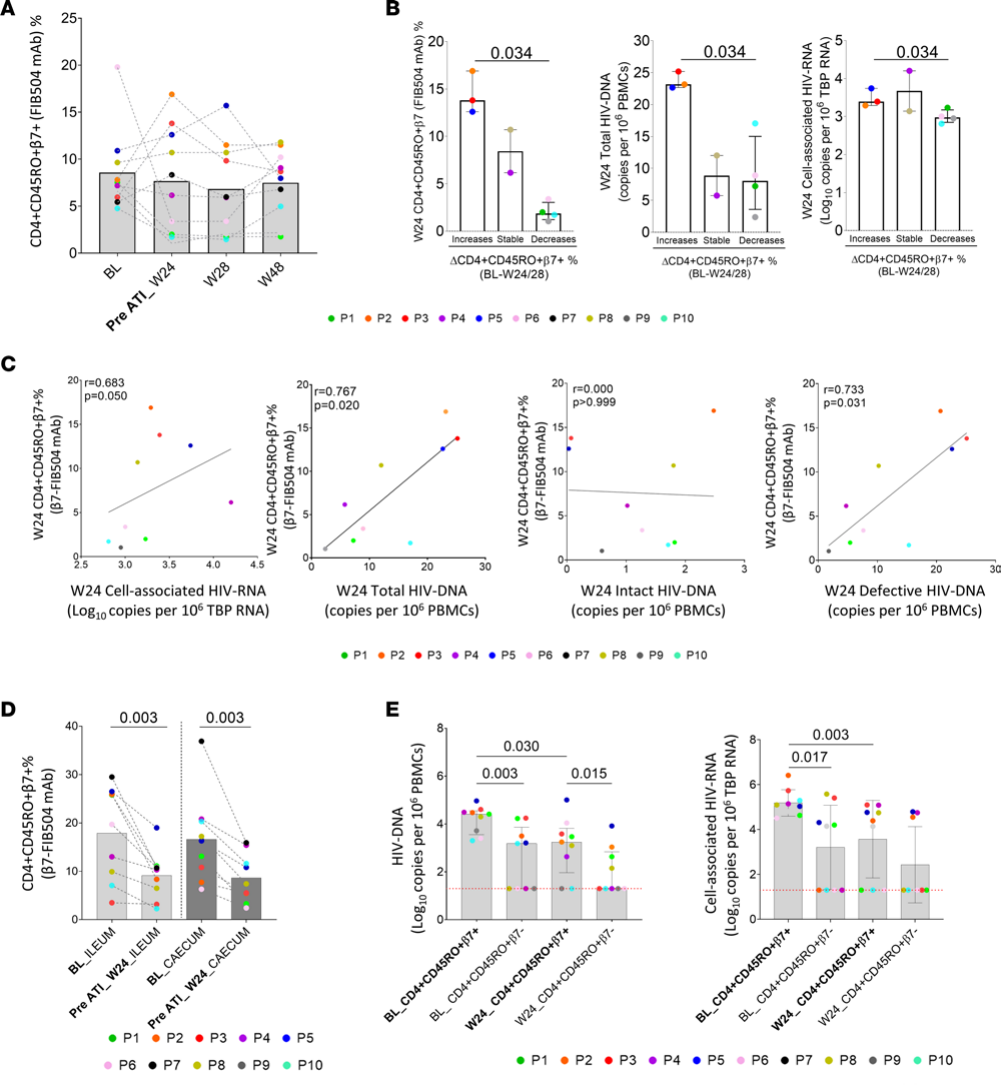
Analysis of the dynamic of β7 expression levels and association with the size of the HIV-1 reservoir at week 24. (**A**) Dynamic of α4β7 expression on CD4^+^ T cells during the follow-up period in PBMCs. (**B**) Correlation between dynamic patterns of peripheral CD4^+^α4β7^+^ T cell levels at week 24 or 28 and memory CD4^+^α4β7^+^ levels, total HIV-1 DNA assayed by FLIP-Seq, and HIV-RNA levels at week 24. (**C**) Correlation between peripheral CD4^+^α4β7^+^ T cells and total, intact, and defective HIV-1 DNA assayed by FLIP-Seq at week 24. CD4^+^α4β7^+^ levels were considered to decrease when there was more than 2.5-fold reduction at week 24 or 28 compared with BL. CD4^+^α4β7^+^ levels were considered to increase when there was more than 1.3-fold change at week 24 or 28 compared with BL. (**D**) Dynamic of α4β7 expression on CD4^+^ T cells in ileum and cecum at BL and week 24. (**E**) Total cell-associated HIV-1 DNA and RNA in peripheral CD4^+^ T α4β7^+^ and α4β7^–^ sorted cells at BL and week 24. Horizontal red line indicates the limit of detection. *P* values were computed using Wilcoxon’s, Mann-Whitney *U*, and Spearman’s test. *P* values are shown within the graphs. BL, baseline; W, week; ATI, analytic treatment interruption.

The percentage of memory CD4^+^ T cells expressing β7 integrin was determined throughout the follow-up period. Quantification of α4β7^+^ levels was performed by gating CD4^+^CD45RO^+^β7^+^ as previously described (9, 14, 31, 32). We did not observe differences in either in the percentage (Figure 4A) or in the absolute numbers (Supplemental Figure 3A) of CD4^+^CD45RO^+^β7^+^ cells in PBMCs during follow-up. Nevertheless, PLWH who restarted ART had higher levels of CD4^+^CD45RO^+^β7^+^ in PBMCs at week 24 compared with those participants who completed the ATI period (Supplemental Figure 4A). The same trend was observed for absolute CD4^+^CD45RO^+^β7^+^ cell counts but at not significant level (Supplemental Figure 5A). Interestingly, those participants who resume ART after ATI increased CD4^+^CD45RO^+^β7^+^ in PBMCs at week 24 or 28, and these increases were associated with nonsignificant higher pVL levels (*P* = 0.077), total cell-associated HIV-1 DNA (*P* = 0.034) and HIV-1 RNA levels (*P* = 0.034) in PBMCs (Supplemental Figure 4B) and higher HIV-1 RNA in ileum (*P* = 0.034) and HIV-1 DNA in cecum at BL (*P* = 0.077) (Supplemental Figure 4C). Likewise, at week 24, those participants who increased CD4^+^CD45RO^+^β7^+^ levels in PBMCs at week 24 or 28 had higher CD4^+^CD45RO^+^β7^+,^ total and defective HIV-1 DNA and HIV-1 RNA levels in PBMCs at just before ATI (week 24) (Figure 4B and Supplemental Figure 4D). The same results were observed when analyzing absolute CD4^+^CD45RO^+^β7^+^ T cell counts (Supplemental Figure 3B and Supplemental Figure 5, B–D). In addition, cell-associated HIV-1 RNA, total and defective, but not intact HIV-1 DNA levels were also directly associated with CD4^+^CD45RO^+^β7^+^ in PBMCs at week 24 (Figure 4C). Furthermore, PLWH who restarted ART presented higher levels of defective HIV-1 DNA levels (Supplemental Figure 4E). Unlike PBMCs (Figure 4A), the CD4^+^CD45RO^+^β7^+^ subset was significant decreased in ileum and cecum at week 24 respect to BL (Figure 4D). There were no decreases in total CD4^+^ T cell levels in gastrointestinal (GI) tissue (Supplemental Figure 4F) and no differences were detected between PLWH who restarted ART and those who did not in GI tissue at BL and week 24 (Supplemental Figure 4G).

To deeply analyze the importance of α4β7 integrin in the HIV reservoir levels, we also determined the HIV-1 reservoir in peripheral CD4^+^CD45RO^+^β7^+^ and β7^–^ sorted cells (Figure 4E). CD4^+^CD45RO^+^β7^+^ cells presented higher levels of total HIV-1 DNA and cell-associated HIV-1 RNA at BL and week 24 than CD4^+^CD45RA^+^β7^–^ cells. Although statistical differences were not observed at week 24 in HIV-1 RNA levels, 33.3% were positive for HIV-1 RNA levels in CD4^+^CD45RA^+^β7^–^ cells compared with 66.6% in CD4^+^CD45RA^+^β7^+^ cells (Figure 4E). Interestingly, we only observed a decrease in HIV-1 DNA and RNA in CD4^+^CD45RO^+^β7^+^ cells at week 24 relative to BL (Figure 4E).

### Inefficient α4β7 blocking in GIT is associated with higher HIV-1 reservoir levels.

**Figure 5:**
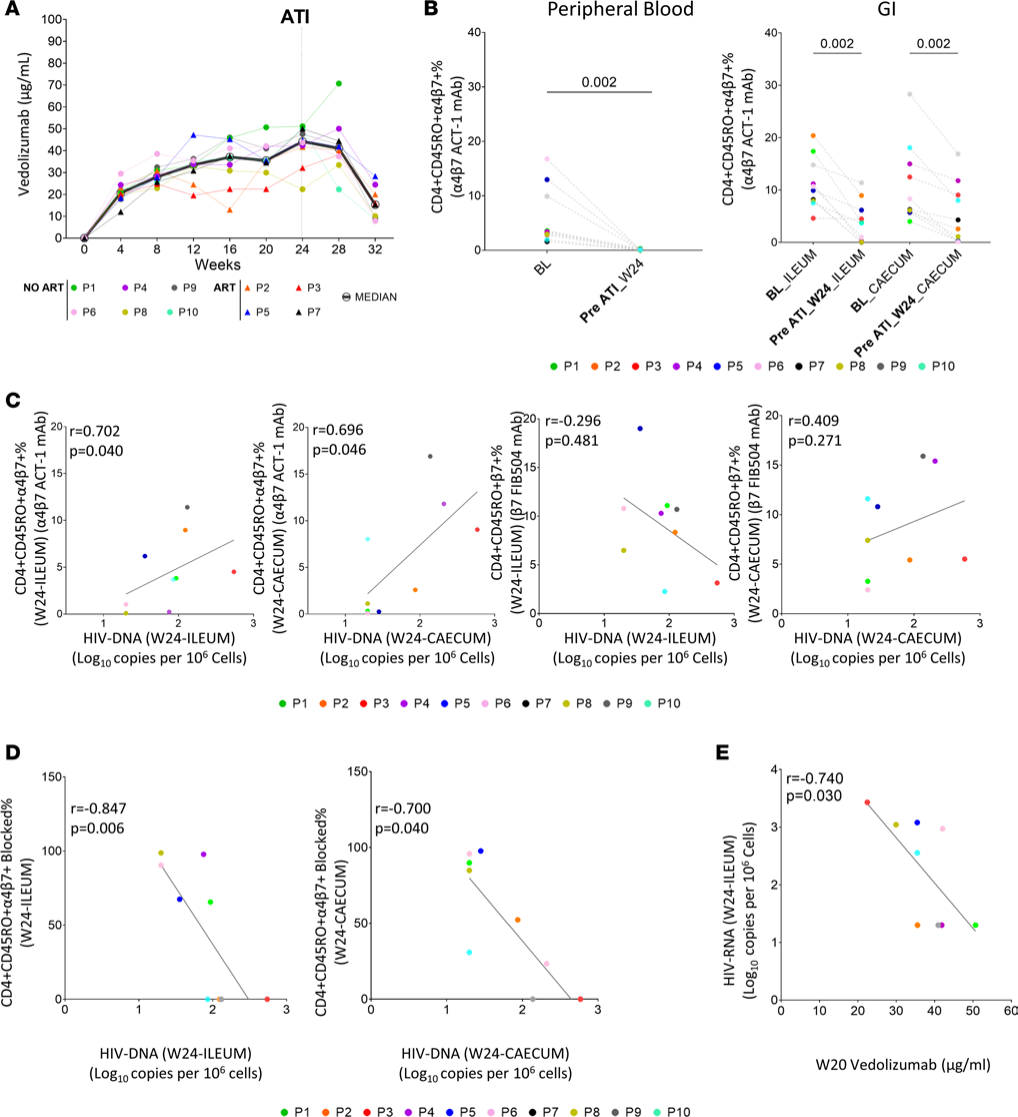
Inefficient α4β7 blocking in GITs is associated with HIV-1 reservoir levels. (**A**) Serum concentration of vedolizumab during the follow-up period. (**B**) Percentage of α4β7 integrin on peripheral CD4^+^ T cells and on ileum and cecum CD4^+^ T cells at BL and week 24. (**C**) Association between α4β7 expression on CD4^+^ T cells and HIV-1 reservoir in ileum and cecum before ATI (week 24). (**D**) Correlation between the percentage of α4β7 integrin blocked by vedolizumab and HIV-1 DNA reservoir in ileum and cecum. (**E**) Correlation between serum concentration of vedolizumab and HIV-1 RNA on ileum before ATI (week 24). *P* values were computed using Wilcoxon’s, Mann-Whitney U, and Spearman’s test. *P* values are shown within the graphs. BL, baseline; W, week; ATI, analytic treatment interruption.

Serum concentrations of vedolizumab were determined prior to each monthly infusion and at weeks 28 and 32 (Figure 5A). The concentrations were similar to those reported in clinical trials of inflammatory bowel disease (20, 21), but the median concentration was slightly lower compared with the clinical trial performed in chronic HIV-1 infection (23). This may occur because vedolizumab can also be bound to the α4β7 integrin present on free virus envelope from participants with high detectable viremia. Using the anti-α4β7 mAb clone ACT-1, with the same target epitope of vedolizumab, we observed that α4β7 integrin was completely blocked by vedolizumab on peripheral CD4^+^ T cells at week 24 (Figure 5B, left), while partial blocking was found in ileum and cecum in the same time point (Figure 5B, right). Indeed, there was a positive correlation between the fraction of CD4^+^CD45RO^+^α4β7^+^ cells not blocked by vedolizumab and HIV-1 DNA in ileum and cecum (Figure 5C, left). However, when we used the clone FIB504, which epitope is recognized independently of bounded vedolizumab, we did not observe this correlation (Figure 5C, right). Taking this into account, we calculated the percentage of blocked α4β7 with the combination of ACT-1 and FIB504 clones. There were no differences in the percentage of CD4^+^CD45RO^+^α4β7^+^ cells blocked between ileum and cecum at week 24 neither between PLWH who restarted ART or those who not (Supplemental Figure 6A). Interestingly, we found an association between HIV-1 DNA reservoir and CD4^+^CD45RO^+^α4β7^+^ cells blocked in both ileum and cecum at the same time point (Figure 5D). Importantly, we also observed a negative correlation between the HIV-1 RNA levels in ileum and vedolizumab concentration at week 20 (Figure 5E); this correlation was also observed for HIV-1 DNA levels on PBMCs (Supplemental Figure 6B).

### Immune checkpoint molecules are associated with α4β7 integrin and HIV-1 reservoir levels.

**Figure 6:**
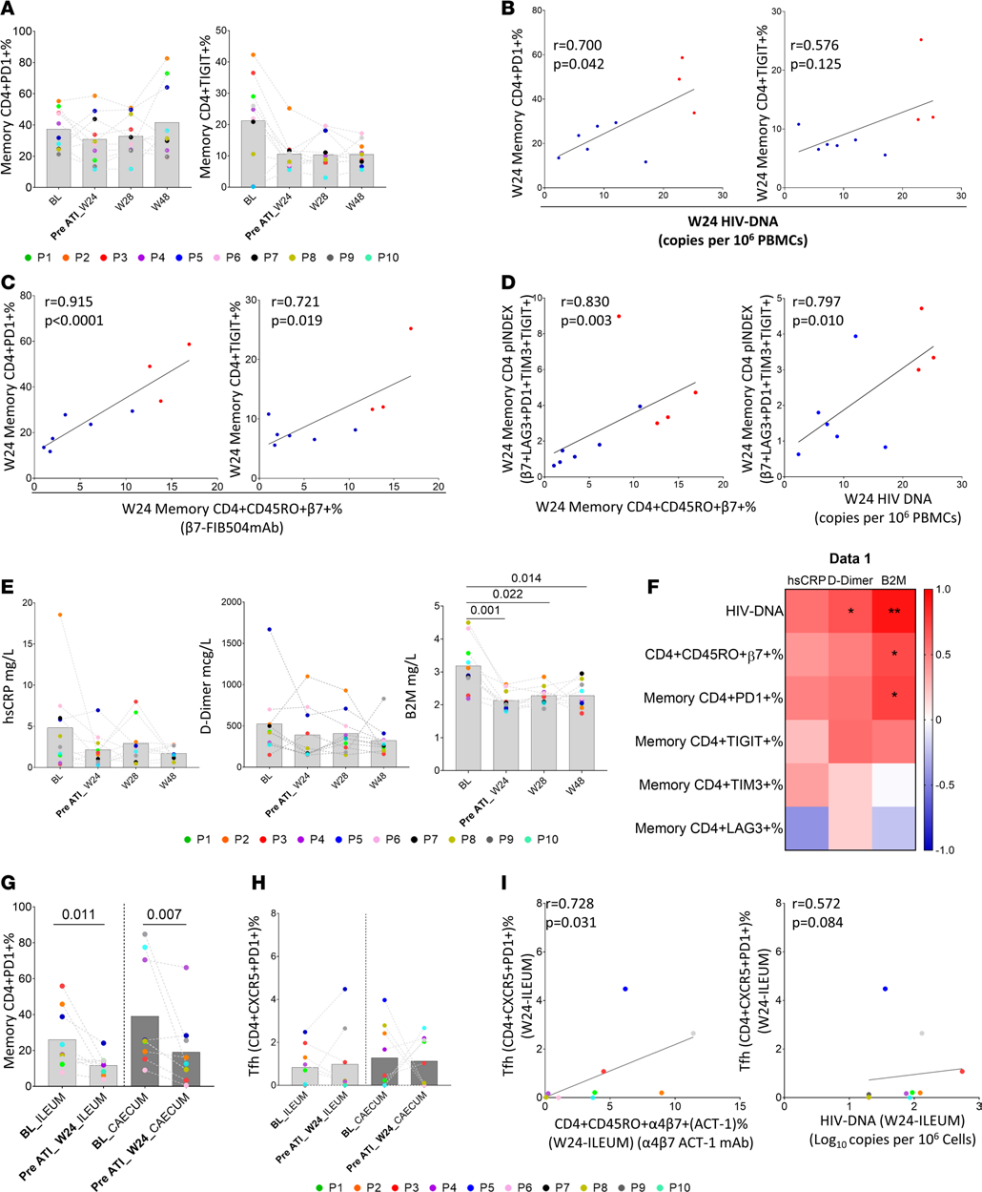
Immune checkpoint molecules are associated with α4β7 and HIV-1 reservoir levels. (**A**) Dynamic of PD1 and TIGIT expression on CD4^+^ T cells during the follow-up period in PBMCs. (**B**) Correlation between total HIV-1 DNA levels assayed by FLIP-Seq and the expression of PD1 and TIGIT on peripheral CD4^+^ T cells before ATI. (**C**) Correlation between the expression of α4β7 integrin and immune checkpoint molecules (PD1 and TIGIT) on peripheral CD4^+^T cells before ATI. (**D**) Correlation between the expression of α4β7 integrin and total HIV-1 DNA in PBMCs assayed by FLIP-Seq and the simultaneous expression of α4β7, LAG3, PD1, and TIM3 on peripheral CD4^+^ T cells just before ATI. (**E**) Plasma soluble biomarkers levels, hsCRP, D-dimer, and B2M during the follow-up period. (**F**) Correlation matrix representing negative (blue shading) and positive (red shading) association between soluble biomarkers and HIV-1 DNA in PBMCs and the expression of α4β7 and immune checkpoint molecules on CD4^+^ T cells. (**G**) Dynamic of PD1 expression on CD4^+^ T cells in ileum and cecum at BL and before ATI (week 24). (**H**) Dynamic of CD4^+^ Tfh cells in ileum and cecum at BL and before ATI (week 24). (**I**) Association between Tfh cells and CD4^+^α4β7^+^ T cells and HIV-1 DNA at ileum before ATI (week 24). *P* values were computed using Friedman’s test with Dunn’s multiple comparisons test correction, Wilcoxon’s, and Spearman’s test. *P* values are shown within the graphs. BL, baseline; W, week; ATI, analytic treatment interruption; Tfh, T follicular helper cells.

Immune checkpoint molecules have been associated with HIV-1 reservoir levels (27). We quantified the expression of PD1, TIGIT, TIM3, and LAG3 in memory CD4^+^ T cells in PBMCs and GI tissue cells and analyzed its association with α4β7 integrin and HIV-1 reservoir levels. Following the same trend as overall α4β7 expression in peripheral blood (Figure 4A), we did not observe differences in either PD1 and TIGIT expression (Figure 6A) or LAG3 and TIM3 (Supplemental Figure 7A) during follow-up. We observed that PD1 memory CD4^+^ T cell levels positively correlated with peripheral total HIV-1 DNA, and a similar but nonsignificant (*P* = 0.125) correlation was observed for TIGIT memory CD4^+^ T cell levels (Figure 6B). In the same way, PD1 and TIGIT memory CD4^+^ T cell levels positively correlated with CD4^+^CD45RO^+^β7^+^ levels (Figure 6C). We calculated the “multiple immune checkpoint phenotype” in combination with β7 integrin (simultaneous expressions of 3 or more of the analyzed markers). The simultaneous expression index of these markers (β7^+^LAG3^+^PD1^+^TIM3^+^TIGIT^+^) positively correlated with CD4^+^CD45RO^+^β7^+^ and peripheral total HIV-1 DNA (Figure 6D), showing the highest levels in PLWH who restarted ART after ATI (Figure 6D and Supplemental Figure 7B). Furthermore, we analyzed whether these multiple immune checkpoints, α4β7^+^ expression, and HIV-1 reservoir were associated with inflammation. Inflammatory soluble markers, such as high-sensitivity C-reactive protein (hsCRP), the coagulation biomarker D-dimer (DD), and β2 microglobulin (B2M), were assayed (Figure 6E). B2M levels decreased during the follow-up period (Figure 6E, right) and at week 24 were associated with α4β7 and PD1 memory CD4^+^ T cell expression and with HIV-1 DNA levels, which in turn were also associated with DD levels (Figure 6F).

Finally, we also analyzed these molecules in GIT. In this case, the HLA-DR, LAG3, TIM3 (Supplemental Figure 7C), and PD1 expression (Figure 6G) were significantly decreased in memory CD4^+^ T cells at week 24 with respect to BL in ileum and cecum, contrary to what occurred in peripheral blood (Figure 6A). Follicular CD4^+^ T (Tfh) cells express PD1 and are enriched in α4β7 integrin (33). Although Tfh cells levels did not change during follow-up (Figure 6H), at week 24, Tfh cells levels were positively associated with the fraction of CD4^+^α4β7^+^ not blocked by vedolizumab, and a nonsignificant positive correlation was found with total HIV-1 DNA in GIT (Figure 6I).

### Retinoic acid is associated with reservoir levels in GIT.

**Figure 7:**
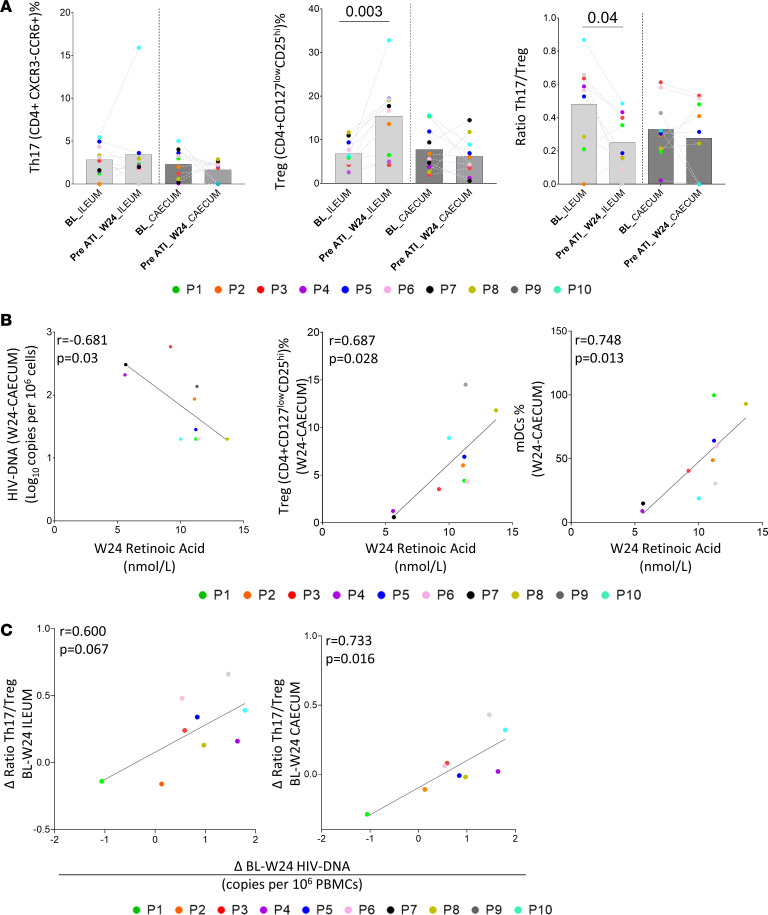
Retinoic acid plasma levels are associated with reservoir levels in GITs. (**A**) Dynamic of Th17 cells, Tregs, and Th17 cell/Treg ratio in ileum and cecum during the follow-up period. (**B**) Association between retinoic acid plasma levels and HIV-1 DNA, Tregs, and myeloid dendritic cells (mDCs) at cecum before ATI (week 24). (**C**) Direct association between the dynamic of HIV-1 DNA reservoir in PBMCs and the Th17 cell/Treg ratio in ileum and cecum before ATI (week 24). *P* values were computed using Wilcoxon’s and Spearman’s test. *P* values are shown within the graphs. BL, baseline; W, week; ATI, analytic treatment interruption; Th17, IL-17–producing T helper cells.

The main GIT cell subsets associated with higher α4β7 integrin expression are Tfh cells, regulatory CD4^+^ T cells (Tregs), and IL-17–producing T helper (Th17) cells. However, we did not observe associations between Treg and Th17 cell levels and α4β7 expression, in contrast to Tfh cells (Figure 6I). Dendritic cells are the major producers of retinoic acid, which is required for inducing gut-tropic lymphocytes. Retinoic acid potentiates the induction of gut homing FoxP3^+^ Tregs and inhibits the development of Th17 cells. Th17 cell/Treg ratio and retinoic acid are involved in the maintenance of GIT homeostasis and damage (34). We found that Treg levels were significant increased, and consequently, the ratio Th17 cell/Treg decreased at week 24 in ileum (Figure 7A). Although we did not observe differences in retinoic acid plasma levels during follow-up (Supplemental Figure 8A), a negative correlation between total HIV-1 DNA levels in cecum and retinoic acid and a positive association between Tregs and myeloid dendritic cells in cecum with retinoic acid levels were observed at week 24 (Figure 7B). Finally, changes between peripheral HIV-1 DNA reservoir levels between BL and week 24 (Supplemental Figure 8B) and the Th17 cell/Treg ratio in ileum and cecum showed a positive nonsignificant and significant association, respectively(Figure 7C).

## Discussion

In this clinical trial, we analyzed the safety and efficacy of vedolizumab combined with ART to achieve virological remission in treatment-naive recently infected PLWH after ATI. Our results show that vedolizumab was safe and well tolerated. Nevertheless, no sustained undetectable viremia was seen during the ATI period. However, using this model we unveiled important insights about the role of α4β7 expression in HIV-1 reservoir levels in peripheral blood and GIT in humans.

A previous study performed in individuals with chronic HIV-1 infection (23), using a similar regimen of vedolizumab to the one used in our study, also showed that vedolizumab was well tolerated, confirming a safe spectrum profile in PLWH. In the same study, vedolizumab was also not able to induce virological remission after ART interruption (23), in accordance with previous findings in the SIV model (24–26). However, the criteria for ART reintroduction after ATI in our study allowed us to observe that 60% of participants completed the ATI with no decreased CD4^+^ T cell levels, and pVL at the end of the ATI period ranged from 1,590 to 36,950 HIV-1 RNA copies/mL (median [IQR]; 5,495 HIV-1 RNA copies/mL [3,311–13,804 HIV-1 RNA copies/mL]). Interestingly, the proportion of participants off ART and the time to achieve more than 1,000 HIV-1 RNA copies/mL was higher compared with that in an historical control group (28), especially when participants with protective alleles were removed from the analysis, as a potential confounding factor. It is important to note that these differences were observed despite the less favorable profile of the vedolizumab group in terms of the lower time on suppressive ART and the trend to have higher pre-RT pVL, both factors associated with a faster viral recrudescence and higher levels of viremia after ATI (35, 36). Despite this modest efficacy effect, these data support the further testing of vedolizumab in combination with other immunotherapies for HIV-cure strategies.

Our unique clinical trial design allowed us to analyze the role of α4β7 expression on peripheral blood and tissue and its effects on HIV-1 reservoir levels after ART initiation in humans. First, we analyzed HIV-1 reservoir dynamics, cell-associated HIV-1 DNA and RNA, on PBMCs and GITs during the follow-up period. As expected, there was a fast decrease of HIV-1 reservoir in peripheral blood during the first 24 weeks after ART initiation, as it has been previously described after early ART and in contrast to what has been observed in chronically ART-suppressed individuals (37–39). In our clinical trial, study participants who resumed ART early during the ATI (*n* = 4) showed higher levels of HIV-1 reservoir, total cell-associated HIV-1 DNA and RNA, at study entry and at ATI start in contrast to participants who remained off ART up to week 48 (*n* = 6) in which peripheral HIV-1 DNA levels remained lower along the study. Similarly, low viral reservoir, total cell-associated HIV-1 DNA and RNA, has been previously reported to be associated with a longer time to viral rebound (35, 36). Little is known about HIV-1 reservoir dynamics in gut-associated lymphoid tissue after early ART initiation (40), owing to the difficulty of obtaining gut biopsies in PLWH during acute HIV-1 infection (41). In our study, we also observed a sharp decrease of the HIV-1 DNA levels in GITs as it occurred in PBMCs; however, the strong direct association between pre-ART pVL and HIV-1 DNA levels in GITs, but not with HIV-1 DNA in peripheral blood, highlights the important contribution of tissue reservoir to viremia, as suggested in animal models (42).

The association of α4β7 levels and blocking with the modulation of HIV-1 reservoir landscape in peripheral blood and tissue in humans remains unclear. Our results revealed strong associations between memory CD4^+^α4β7^+^ and HIV-1 reservoir levels (both, cell-associated HIV-1 DNA and HIV-1 RNA) in PBMCs and in 2 GITs locations, ileum and cecum. These results are similar to those found in a cohort of PLWH who started ART during primary infection, where total HIV-1 DNA was directly associated with α4β7 expression in intestinal lamina propria mononuclear cells of ileum and rectum (43). Additionally, we were able to distinguish that this association of α4β7 levels with peripheral reservoir was mainly due to defective provirus and not because of the intact proviral reservoir (44). However, the clinical relevance of defective HIV-1 DNA levels came from the fact that these levels were associated with further ART reintroduction after ATI. Further insights into the role of α4β7 expression on HIV-1 reservoir establishment came from the different α4β7 expression kinetics in peripheral blood and tissue. It is known that memory CD4^+^α4β7^+^ cells are an early target of HIV-1 infection following mucosal transmission (13, 14, 45, 46). We found that overall α4β7 expression on peripheral CD4^+^ T cells did not change during combined treatment with ART for 24 weeks. However, a detailed analysis of the dynamics of α4β7 expression on peripheral CD4^+^ T cells demonstrated that those participants with decreased CD4^+^α4β7^+^ cells before ATI achieved the lowest levels, and this was associated with no recrudescent of viral rebound after ATI and at the same time with lower total and defective HIV-1 DNA and HIV-1 RNA levels. These results are important because based on α4β7 dynamics and levels before ATI, we may predict those individuals who are going to resume ART. Regarding GI tissue, a uniform downregulation of α4β7 expression was observed on ileum and cecum CD4^+^ T cells during follow-up. To investigate the α4β7 block, we used two different antibodies sharing or not the same epitopes of vedolizumab binding site. This strategy led us to uncover that anti-α4β7 treatment completely blocks α4β7 in the periphery but not in GI tissue. Interestingly, we found that the cell-associated HIV-1 DNA was strongly associated with the percentage of α4β7 not blocked on GITs memory CD4^+^ T cells but not with total α4β7 expression. These data were supported by the higher HIV-1 reservoir levels, cell-associated HIV-1 DNA and HIV-1 RNA, in sorted α4β7^+^ peripheral blood CD4^+^ T cells compared with α4β7^–^ cells in accordance with previous findings in the simian model (13) and in humans in cells positive for α4β1 heterodimer that were enriched in HIV-1 content compared with α4β1^–^ cells (47). These results also open the question of whether vedolizumab administration at higher doses would have increased virological efficacy. In this sense, it is important to note the favorable safety profile of vedolizumab compared with other immunomodulators for the development of adverse events, such us progressive multifocal leukoencephalopathy (48, 49). In our study, participants received monthly doses of 300 mg vedolizumab (4.3 mg/kg [3.6–5.02 mg/kg]) together with ART, the approved dose used for the treatment of inflammatory bowel disease (20, 21). In previous studies, the primatized analog of anti-α4β7 was administered at a dose of 50 mg/kg, 10-fold higher than the dose in the present study, fully masking the expression of α4β7 expressed on the surface of lymphocytes harvested from GITs biopsies (17, 18, 50, 51). These results suggest that the reduction of HIV-1 reservoir may be associated with vedolizumab concentration. Indeed, we found an inverse correlation between total cell-associated HIV-1 DNA and HIV-1 RNA in peripheral blood and ileum, respectively, with vedolizumab levels just before ATI.

Afterward, we performed additional phenotypical characterization of α4β7^+^ CD4^+^ T cells and analyzed their association with HIV-1 reservoir levels. The expression of the immune checkpoint molecules PD1, LAG3, and TIM3 on T cells was also previously identified as a preferential niche for the HIV-1 reservoir enrichment (27). In accordance with previous studies (52), we found that the coexpressing phenotypes of these immune checkpoint molecules and α4β7 expression on memory CD4^+^ T cells exhibited strong correlations with total cell-associated HIV-1 DNA. These immune checkpoint molecules were identified as a strong predictor of time to viral rebound in some ATI cohorts (53). In our clinical trial, study participants who restarted ART exhibited higher levels of memory CD4^+^α4β7^+^LAG3^+^PD1^+^TIGIT^+^TIM3^+^ T cells at the ATI time point. Interestingly, we found that this immune checkpoint molecule and α4β7^+^ phenotype were associated with inflammatory biomarkers, such as β2M and DD levels, previously related with cell-associated HIV-1 RNA (54). Besides, we found a direct association between total cell-associated HIV-1 DNA and DD and β2M levels in plasma. These results suggest a connection between HIV-1 reservoir and inflammatory parameters, potentially related with the T cell turnover induced by the virus and the β2M shedding even in PLWH on treatment. Remarkably, we found decreased levels of LAG3, TIM3, and PD1 CD4^+^ T cell in tissue during the follow-up, reflecting the decrease HIV-1 reservoir in tissue.

Finally, we analyzed immune reconstitution in GITs of the 3 main functional subsets of CD4^+^ T cells that express α4β7, Tregs, Tfh cells, and Th17 cells (33, 55), in relation to GIT homeostasis and HIV-1 reservoir. No reconstitution was observed in Th17 and Tfh cells. Indeed, Tfh cells, which constitutively express PD1, were associated with free α4β7 levels, not blocked by vedolizumab, with a trend toward increased HIV-1 reservoir in ileum, suggesting the preferential infection of this T cell subset (56). Conversely, we did find increased Treg levels in ileum during the follow-up, and, subsequently the Th17 cell/Treg ratio decreased, which has been associated with GIT homeostasis and disease progression (57, 58). Additionally, we observed that the Th17 cell/Treg ratio was associated with HIV-1 DNA reservoir changes in the periphery during the follow-up period. Besides, retinoic acid, produced by dendritic cells, plays an essential role in gut homeostasis and induces the expression of α4β7 (59, 60). Furthermore, dendritic cells from GITs enhance Tregs’ differentiation in a retinoic acid–dependent manner (61) as well as convert vitamin A in retinoic acid (62). In agreement with this, our results show a direct association between retinoic acid plasma levels and myeloid dendritic and Treg levels in cecum tissue.

This differential immune reconstitution, depending on GIT location, was concomitant with an inverse correlation of retinoic acid plasma levels with total proviral HIV-1 DNA reservoir in the cecum at week 24. This may support the potential role of retinoic acid as a latency-reversing agent (63).

Overall, our results are in agreement with those of the simian model, in which blocking α4β7 with vedolizumab together with the use of a broadly neutralizing antibody delayed viral rebound after ATI (51), but also those in humans, in which the use of anti-α4β7 therapy was associated with the attrition of lymphoid aggregates that may potentially affect HIV-1 reservoir levels in GIT (64).

One of the major limitations of this study was the low number of participants and that most of them were men. However, the stringent inclusion criteria, only participants with confirmed acute/recent HIV-1 infection were included, and the extensive tissue sampling requirements justified the trial sample size and sex bias. Another limitation was the lack of a randomized control group. However, we were able to compare the ATI outcomes from our study with the placebo recipients from a recently reported study performed in a very comparable population (28).

In conclusion, vedolizumab, administered for 24 weeks, was safe and well tolerated in PLWH treated soon after HIV infection. No sustained virological remission after ART interruption was found in participants treated with vedolizumab. Importantly, this clinical trial suggests that α4β7 is an important determinant of HIV-1 reservoir levels seeding in peripheral blood and specially in tissues in humans and therefore, supports further testing of vedolizumab in combination with other compounds as a promising tool for HIV-1 cure strategies.

## Methods

### Sex as a biological variable.

Cisgender woman and men were included in the study.

### Study design.

This was an open-label, single-arm phase II clinical trial to assess the safety and virological effect of vedolizumab (Entyvio) and ART in participants with recent HIV-1 infection and naive for ART who underwent ATI (Figure 1). Commercially available vedolizumab and ART were supplied by Virgen del Rocío University Hospital. The ART regimen was dolutegravir (50 mg), tenofovir alafenamide (25 mg), and emtricitabine (200 mg), all qd. The clinical trial was performed at the Clinic Unit of Infectious Diseases, Microbiology and Parasitology, and at the phase I/II Clinical Trials Units at Virgen del Rocío University Hospital. PLWH were eligible if they were 18 to 65 years of age. Participants were required to have a CD4^+^ T cells count of more than 350 cells/μL and a viremia more than 10^4^ HIV-1 RNA copies/mL. Study participants were recruited between September 2018 and June 2019 and started ART together with 300 mg vedolizumab intravenous infusions at 0, 4, 8, 12, 16, 20, and 24 weeks. At week 24 of follow-up ART and vedolizumab treatment were interrupted. Biopsies from ileum and cecum were obtained at BL and week 24, before ART and before ATI, respectively. Throughout the treatment interruption phase, participants were monitored monthly by measuring CD4^+^ T cell counts and plasma viremia. Criteria to restart ART during the ATI were a decrease in the levels of CD4^+^ T cells below 350 cell/μL or pVL levels above 10^5^ HIV-1 RNA copies/mL (2 consecutive measurements 1 month apart). These nonstringent restarting ART criteria were chosen to avoid missing a potential control of HIV-1 replication after a potential peak of viremia after ATI. Participants who reached week 48 of follow-up without meeting restart criteria were advised to restart ART if they had detectable plasma viremia (>20 HIV-1 RNA copies/mL).

The safety endpoint was the proportion of participants with vedolizumab treatment–related adverse events and its severity. All adverse events, severity, and relationships to study product during vedolizumab infusion and follow-up were reported according to the Division of AIDS Table for Grading the Severity of Adult and Pediatric Adverse Events, version 2.0, November 2014. The virological endpoint was defined as the number of participants remaining off ART and who achieved undetectable pVL at week 48 according to the criteria mentioned above.

For post hoc efficacy analysis, we compared this group of participants (the vedolizumab group) with historical controls matched by age, sex, and time of infection, corresponding to the placebo arm of the AELIX-002 (NCT03204617) vaccine trial performed in early-treated PLWH that also included an ATI (28) for 24 weeks using the same ART resumption criteria as the vedolizumab group.

### Laboratory methods.

Absolute CD4^+^ and CD8^+^ T cell counts were measured using an FC500 Flow Cytometer (Beckman-Coulter). The plasma HIV-1 RNA concentration was measured by quantitative PCR (COBAS Ampliprep/COBAS Taqman HIV-1 Test, Roche Molecular Systems; lower detection limit of 20 HIV-1 RNA copies/mL) according to the manufacturer’s protocol.

### PBMC isolation.

PBMCs were isolated using BD Vacutainer CPT Mononuclear Cell Preparation Tubes (with Sodium Heparin) by density gradient centrifugation 1 week before each vedolizumab infusion before ATI and at weeks 28, 32, 36, 40, 44, and 48 of follow-up. PBMCs were cryopreserved in liquid nitrogen until further use.

### Isolation of GI cells.

Ileal and cecal biopsies were obtained during colonoscopy at BL and at ATI start (week 24). These 2 locations were biopsied for having a representation of immune inductive and effector sites, respectively (65). Fresh biopsies (10–13 pieces) were transported in R10 medium (RPMI medium supplemented with 10 % FBS, 1% penicillin, and 1% L-glutamine) and processed immediately. Intestinal biopsies were washed with PBS and 14% ethylene diamine tetra-acetic acid (EDTA) for 30 minutes at 37°C in agitation. The biopsies were then physically disrupted with blades. Next, the intestinal biopsies were transferred to 20 mL R10 containing 20 mg Type IV collagenase (Sigma-Aldrich) and incubated for 30 minutes at 37°C with gentle agitation. After the first 15-minute round of incubation with collagenase solution, biopsies were physically disrupted by syringes with needles. The disrupted tissue was transferred into the R10-collagenase solution for a second round consisting of a 15-minute incubation in gentle agitation. After incubation, single-cell suspension was obtained by filtering through a 70 μm cell strainer and washed with R10 medium. Cells were cryopreserved in liquid nitrogen until further use. Two biopsies’ pieces were frozen intact in RNA-later and snap frozen at –80°C for further RNA and DNA extraction.

### Assay of soluble biomarkers and plasma levels of retinoic acid.

Serum and plasma samples were collected in serum separation tubes and in EDTA tubes and stored at –20°C until subsequent analysis of the following biomarkers: hsCRP, β2M, and DD. The levels of hsCRP and β2M were determined by an immunoturbidimetric serum assay using a Cobas 701 analyzer (Roche Diagnostics). DD levels were measured by an automated latex-enhanced immunoassay (HemosIL D-dimer HS 500; Instrumentation Laboratory). Retinoic acid plasma levels were determined by UHPLC-MS/MS according to previously described method (66–68). All the assays were performed following the manufacturers’ instructions.

### Plasma levels of vedolizumab and immunogenicity.

Serum concentrations of vedolizumab and the presence of antidrug antibodies were determined in serum samples using the ELISA RIDASCREE VDZ Monitoring (r-biopharm). The assays were performed following the manufacturer’s instructions.

### Immunophenotyping and quantification of α4β7 cells.

Cryopreserved PBMCs were thawed, washed (650*g*, 5 minutes, room temperature) with PBS, and incubated for 35 minutes at room temperature with LIVE/DEAD Fixable Aqua Dead Cell Stain (Life Technologies) and extracellular anti-human antibodies anti-CD45RA (FITC); anti-TIGIT (PerCP-Cy5.5); anti-LAG3 (BV605); anti-PD1 (BV510); anti-integrin β7 (BV711); anti-CD27 (BV786); anti-CD38 (BV650); anti-CD3 (APCH7); anti-integrin α4β7 (APC); anti-TIM3 (PeCF594); anti-HLA-DR (BV570); anti-CD4 (AF 700); and anti-CD19, anti-CD14, and anti-CD56 (Pacific Blue) (See Supplemental Table 3). PBMCs were then washed with PBS and permeabilized with the Fixation/Permeabilization FoxP3 Kit (eBioscience) according to the manufacturer’s instructions. Cells were stained intracellularly at 4°C for 30 minutes with anti-Ki67 (PE) and then washed and fixed in PBS containing 4% paraformaldehyde. Samples were acquired using LSR-II Fortessa Cytometer (BD Immunocytometry Systems), and analyses were performed using FlowJo, version 9.2.

Isolated GI cells were thawed, washed (650*g*, 5 minutes, room temperature) with PBS, and incubated for 35 minutes at room temperature with LIVE/DEAD fixable Violet Dead cell stain and extracellular anti-human antibodies anti-CCR6 (AF 647); anti-CD45RA (FITC); anti-CD25 (PE-Cy7); anti-CXCR5 (BV421); anti-LAG3 (BV605); anti-CXCR3 (PerCP-Cy5.5); anti-PD1 (BV510); anti-CD127 (BUV737); anti-CD45 (BUV805); anti-CD8 (BUV615); anti-CD69 (BB700); anti-CD103 (BV480); anti-CCR7 (BUV563); anti-CD3 (APC-H7); anti-TIM3 (PE/DAZZLE 594); anti-integrin α4β7 (APC); anti-CD123 (Alexa Fluor700); anti-CD11c (BV650); anti-HLA-DR (BV570); anti-integrin β7 (BV711); anti-CD27 (BV786); and anti-CD19, anti-CD14, anti-CD20, and anti-CD56 (Pacific Blue) (See Supplemental Table 3). Cells were then washed and permeabilized using the Fixation/Permeabilization FoxP3 Kit (eBioscience) according to the manufacturer’s instructions. Cells were stained intracellularly at 4°C for 30 minutes with anti-FoxP3 (PE-Cy5) and anti-Ki67 (PerCP-eFluor 710) and then washed and fixed in PBS containing 4% paraformaldehyde. Samples were acquired using a Cytek Aurora Spectral Cytometer 4L (Cytek Biosciences), and analyses were performed using FlowJo, version 9.2.

Anti-integrin α4β7 mAb (APC; clone: ACT-1) was provided by Danlan Wei and James Arthos (National Institute of Allergy and Infectious Disease, NIH, Bethesda, Maryland, USA). Anti-integrin α4β7 mAb (APC; clone: ACT-1) and vedolizumab share the same epitope. Quantification of integrin α4β7 levels was performed using anti-α4β7 mAb (APC; clone: ACT-1) at BL and by gating CD4^+^CD45RO^+^β7^+^ during the follow-up period. Previous studies have demonstrated that CD4^+^CD45RO^+^β7^+^ cells in peripheral blood are more than 99% α4β7^+^ (9, 14, 31, 32); therefore, this gating strategy was used to quantify α4β7 expression on CD4^+^ T cells (Supplemental Figure 9). The percentage of α4β7 integrin blocked by vedolizumab was calculated through the combination of anti-α4β7 (APC; clone ACT-1) and anti-β7 (BV711; clone FIB504).

### Cell sorting.

CD4^+^ memory T cells α4β7^+^ and α4β7^–^ were sorted from PBMCs. Cryopreserved PBMCs were thawed, washed with PBS (650*g*, 5 minutes, room temperature), and incubated for 35 minutes at room temperature with LIVE/DEAD Fixable Violet Dead cell stain and extracellular anti-human antibodies anti-CD45RA (FITC); anti-integrin β7 (BV711); anti-integrin α4β7 (APC); anti-CD27 (BV786); anti-CD3 (APC-H7); anti-CD4 (AF700); and anti-CD19, anti-CD14, and anti-CD56 (Pacific Blue) (See Supplemental Table 3). CD4^+^CD45RO^+^β7^+^ and CD4^+^CD45RO^+^β7^–^ cells were sorted using a BD FACSAria Fusion Flow Cytometer (BD Immunocytometry Systems), and analysis was performed using FlowJo, version 9.2.

### Quantitation of cell-associated HIV-1 DNA and RNA.

The procedures for quantitation of total cell-associated HIV-1 DNA and RNA have been previously described in detail (69). Briefly, levels of total cell-associated HIV-1 DNA and RNA were quantified by droplet-digital PCR (ddPCR) from extracted DNA and RNA using the Bio-Rad QX200 Droplet Reader. Genomic DNA was extracted using the Blood DNA Mini Kit (Omega, Bio-Tek) for the bulk of PBMCs and QIAamp DNA Micro Kit (Qiagen) for CD4^+^CD45RO^+^ β7^+^ and β7^–^ sorted cells following the manufacturer’s protocol. RNA was extracted using NucleoSpin RNA purification kit (Macherey-Nagel) for the bulk of PBMCs and RNeasy Micro Kit (Qiagen) for sorted cells following the manufacturer’s protocol. DNA and RNA concentrations were measured by the Qubit Assay (Thermo Fisher Scientific) and diluted to a concentration of 30 ng/μL. Bio-Rad QX200 ddPCR system was run according to the manufacturer’s protocol, using an annealing temperature of 58°C, using 2 pairs of primers targeting LTR and Gag regions (69). Copy numbers were calculated using Bio-Rad QuantaSoft software v.1.7.4. RPP30 (to cell-associated HIV-1 DNA) and TBP genes (to cell-associated HIV-1 RNA) were the host cell genes used to normalize HIV-1 copies.

### Full-length individual proviral sequencing in PBMCs.

Full-length individual proviral sequencing (FLIP-Seq) was assayed in PBMCs at week 24. Genomic DNA, previously extracted from PBMCs (DNeasy Blood & Tissue kit, QIAGEN), was diluted to single proviral genomes based on ddPCR results and Poisson distribution statistics, where 1 provirus was present in approximately 20%–30% of wells. Subsequently, DNA was subjected to HIV-1 near-full-genome amplification using a single-amplicon nested PCR approach. The reaction was composed of 1 unit of Invitrogen Platinum Taq (catalog 11302-029) per 20 μL of reaction, 1× reaction buffer, 2 mM MgSO_4_, 0.2 mM dNTP, and 0.4 μM of forward (first-round nested PCR: U5-623F, 5′-AAATCTCTAGCAGTGGCGCCCGAACAG-3′; second-round nested PCR: U5-638F, 5′-GCGCCCGAACAGGGACYTGAAARCGAAAG-3′) and reverse primer (first-round nested PCR: U5-601R, 5′-TGAGGGATCTCTAGTTACCAGAGTC-3′; second-round nested PCR: U5-547R, 5′-GCACTCAAGGCAAGCTTTATTGAGGCTTA-3′). The PCR was performed using the following thermocycler program: 2 minutes at 92°C, 10 cycles [10 seconds at 92°C, 30 seconds at 60°C, 10 minutes at 68°C], 20 cycles [10 seconds at 92°C, 30 seconds at 55°C, 10 minutes at 68°C], 10 minutes at 68°C, and 4°C infinite hold. PCR products were visualized by agarose gel electrophoresis. All near-full-length genomes were subjected to Illumina MiSeq sequencing at the MGH DNA Core facility. Large deleterious deletions (<8,000 bp of the amplicon aligned to HXB2), out-of-frame indels, premature/lethal stop codons, internal inversions, or packaging signal deletions (≥15 bp insertions and/or deletions relative to HXB2) were identified by an automated pipeline written in Python programming language (https://github.com/BWH-Lichterfeld-Lab/Intactness-Pipeline, commit ID 0c59371) (70), and the presence or absence of APOBEC-3G/3F-associated hypermutations was determined using a Los Alamos National Laboratory HIV-1 Sequence Database Hypermut 2.0 program (71). Viral sequences without any of the mutations mentioned previously were classified as intact sequences. Phylogenetic distances between sequences were determined through maximum-likelihood trees in MEGA (https://www.megasoftware.net/) and visualized with Highlighter plots (https://www.hiv.lanl.gov/content/sequence/HIGHLIGHT/highlighter_top.html).

### Statistics.

Continuous variables were expressed as medians and IQRs, and categorical variables were expressed as numbers and percentages. Friedman’s test with Dunn’s multiple comparisons test correction was used to assess differences during the follow-up period. Wilcoxon’s signed-rank test was used to analyze related samples and Mann-Whitney *U* and χ^2^ tests were used to analyze differences between groups. Correlations between variables were assessed using Spearman’s rank test. Log-rank test and Kaplan-Meier curves were used for time-to-event analysis regarding virological efficacy compared with the historical control group. All *P* values of less than 0.05 were considered significant. Statistical analysis was performed using Statistical Package for the Social Sciences software (SPSS 22.0). Multiple immune checkpoint phenotypes were constructed using Pestle version 1.6.2 and Spice version 6 (provided by M. Roederer, NIH) and quantified with the polyfunctionality index algorithm (Pindex) employing the 0.1.2 beta version of FunkyCells Boolean Dataminer software, provided by Martin Larson (INSERM U1135, Paris, France) as previously described (72).

### Study approval.

All participants gave written informed consent prior to the study start, The clinical trial was approved by the Seville Provincial Ethics Committee of Research with Medicines (NCT03577782, please visit https://clinicaltrials.gov/ for protocol summary; internal code: FIS-VED-2017-01; study code: no. EudraCT: 2018-000497-30) and authorized by the Spanish Agency for Medicines and Medical Devices (AEMPS).

### Data availability.

Data generated by this study are available in the Supporting Data Values file or upon request to the corresponding author. However, due to the sensitivity of the data and patient confidentiality, individual participant data will not be made available.

## Author contributions

All authors reviewed and approved the submitted version of the manuscript. MRJL and CGC contributed equally to this work — we decided to list the PhD first and the PhD student second. LFLC, PV, NE, and CRO recruited the participants and provided PLWH blood samples. IRJ, AJCB, and RM supervised the clinical trial. SS and MFA performed the biopsies and provided PLWH GIT samples. ERM, MRJL, and CGC designed the experiments. MRJL, CGC, CG, IR, GG, JGSH, RRB, FG, MICS, IG, and AIAR performed the experiments. CB, IM, and BM provided data from the historical control group. MRJL, CGC, and ERM analyzed and interpreted the data and wrote the manuscript. KN, CB, IM, BM, JV, SB, APG, XY, and ML reviewed and contributed to manuscript discussion. ERM conceived the idea, coordinated the project, and acquired funding for the study.

## Supplementary Material

Supplemental data

ICMJE disclosure forms

Supporting data values

## Figures and Tables

**Table 1:**
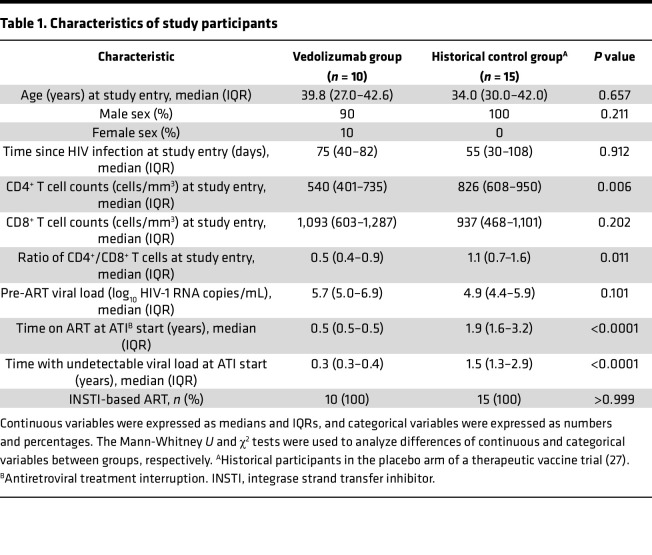
Characteristics of study participants

## Version 1 07/09/2024

In-Press Preview

## Version 2 08/22/2024

Electronic publication
