# Supplementary material for: Vedolizumab and ART in recent HIV-1 infection unveil the role of α4β7 in reservoir size
Source: JCI Insight. 2024 Jul 9;9(16):e182312. doi: 10.1172/jci.insight.182312 (PMC11343594; doi:10.1172/jci.insight.182312)
Supplement: Supplemental data [file jciinsight-9-182312-s172.pdf]

Event	Vedolizumab Group (n=10)
**Adverse event n (%)**	0 (0%)
Colitis	-
Arthralgia	-
Pyrexia	-
Nasopharyngitis	-
Headache	-
Nausea	-
Abdominal pain	-
Upper respiratory tract infection	-
Fatigue	-
Vomiting	-
Back pain	-
**Any serious adverse event n (%)**	0 (0%)
**Any serious infection[a] n (%)**	0 (0%)

Supplementary Table 1. Adverse events during vedolizumab infusions or post-infusion periods.

All adverse and severe events during vedolizumab infusions were meticulously documented along the follow-up according to the Division of AIDS Table for Grading the Severity of Adult and Pediatric Adverse Events, version 2.0, November 2014.

[a]A serious infection was defined as a serious adverse event of infection according to the classification for adverse event reporting in MedDRA.

Supplementary Table 2. Parameters of interest before and during ATI.

Clinical parameter	Vedolizumab (n=10)	Historical Control[a] (n=15)	*p* value
Viral load before ART (HIV-RNA copies/mL)	5.69 [5.04 – 6.85]	4.90 [5.04 – 6.85]	0.101
Time off ART (weeks)	24 [8 – 24]	8 [5 – 20]	0.060
Time to VL peak (weeks)	8 [4 – 13]	4 [4 – 8]	0.124
VL at peak (HIV-RNA copies/mL)	4.89 [4.13 – 5.39]	4.83 [4.34 – 5.29]	0.912
Slope (HIV-RNA copies/mL/weeks)	0.38 [0.21 – 0.72]	0.45 [0.39 – 0.85]	0.222
Last VL at the end of ATI (HIV-RNA copies/mL)	4.28 [3.67 – 5.32]	4.49 [4.06 – 5.29]	0.698
First positive VL after ATI (HIV-RNA copies/mL)	4.59 [2.91 – 5.07]	4.74 [3.37 – 5.08]	0.657
CD4+ T-cell count at ATI start	747 [628 – 1023]	865 [653 – 1172]	0.506
CD4+ T-cell count at the end of ATI	729 [473 – 943]	687 [549 – 832]	0.868

Continuous variables were expressed as medians and interquartile ranges (IQRs). Mann-Whitney U test was used to analyse differences of non-parametric continuous variables between groups. VL: viral load.

[a]Historical participants in the placebo arm of a therapeutic vaccine trial [27].

Supplementary Table 3

Antibodies	CHANNEL	CLONE	SOURCE	IDENTIFIER
Anti-human CD45RA	FITC	L48	BD Biosciences	Cat# 335039
Anti-human TIGIT	PerCP-Cy5,5	A15153G	Biolegend	Cat# 372718
Anti-human PD-1	BV510	EH12.1	BD Biosciences	Cat# 563076
Anti-human LAG3	BV605	11C3C65	Biolegend	Cat# 369324
Anti-human CCR7	BUV563	3D12	BD Biosciences	Cat# 741317
Anti-human HLA-DR	BV570	L243	Biolegend	Cat# 307637
Anti-human Integrin-β7	BV711	FIB504	BD Biosciences	Cat# 744012
Anti-human CD4	AF700	RPA-T4	BD Biosciences	Cat# 557922
Anti-human CD27	BV786	L128	BD Biosciences	Cat# 563327
Anti-human CD38	BV650	HIT2	BD Biosciences	Cat# 740574
Anti-human CD19	Pacific Blue	SJ25-C1	Invitrogen	Cat# MHCD1928
Anti-human CD14	Pacific Blue	TuK4	Invitrogen	Cat# MHCD1428
Anti-human CD56	Pacific Blue	MEM-188	Biolegend	Cat# 304629
Anti-human CD20	Pacific Blue	2H7	Biolegend	Cat# 302328
Anti-human CD3	APC-H7	SK7	BD Biosciences	Cat# 560176
Anti-human Integrin-α4β7	APC	ACT-1	[a]	[a]
Anti-human TIM3	PE/Dazzle 594	F38-2E2	Biolegend	Cat# 345033
Anti-human Ki67	PE	Ki-67	Biolegend	Cat# 350504
Anti-human Ki67	PerCP-eFluor 710	20Raj1	eBioscience	Cat# 46-5699-42
Anti-human CD123	AF700	32703	R&D, San Diego,CA	Cat# FAB301
Anti-human CD11c	BV650	B-ly6	BD Biosciences	Cat# 563404
Anti-human CD25	PE-Cy7	M-A251	Biolegend	Cat# 356108
Anti-human CXCR5	BV421	RF8B2	BD Biosciences	Cat# 562747
Anti-human CXCR3	Pe-Cy5.5	1C6/CXCR3	BD Biosciences	Cat# 560832
Anti-human CD127	BUV737	HIL-7R-M21	BD Biosciences	Cat# 564300
Anti-human CD45	BUV805	HI30	BD Biosciences	Cat# 612891
Anti-human CD8	BUV615	SK1	BD Biosciences	Cat# 612994
Anti-human CD69	BB700	FN50	BD Biosciences	Cat# 747520
Anti-human CD103	BV480	Ber-ACT8	BD Biosciences	Cat# 746472
Anti-human FoxP3	PE-Cy5	236A/E7	Invitrogen	Cat# 15-4777-42

[a]Anti-integrin α4β7 mAb (APC; clone: ACT-1) was kindly provided by Dr. Danlan Wei and Dr. James Arthos, National Institute of Allergy and Infectious Disease (NIAID-NIH).

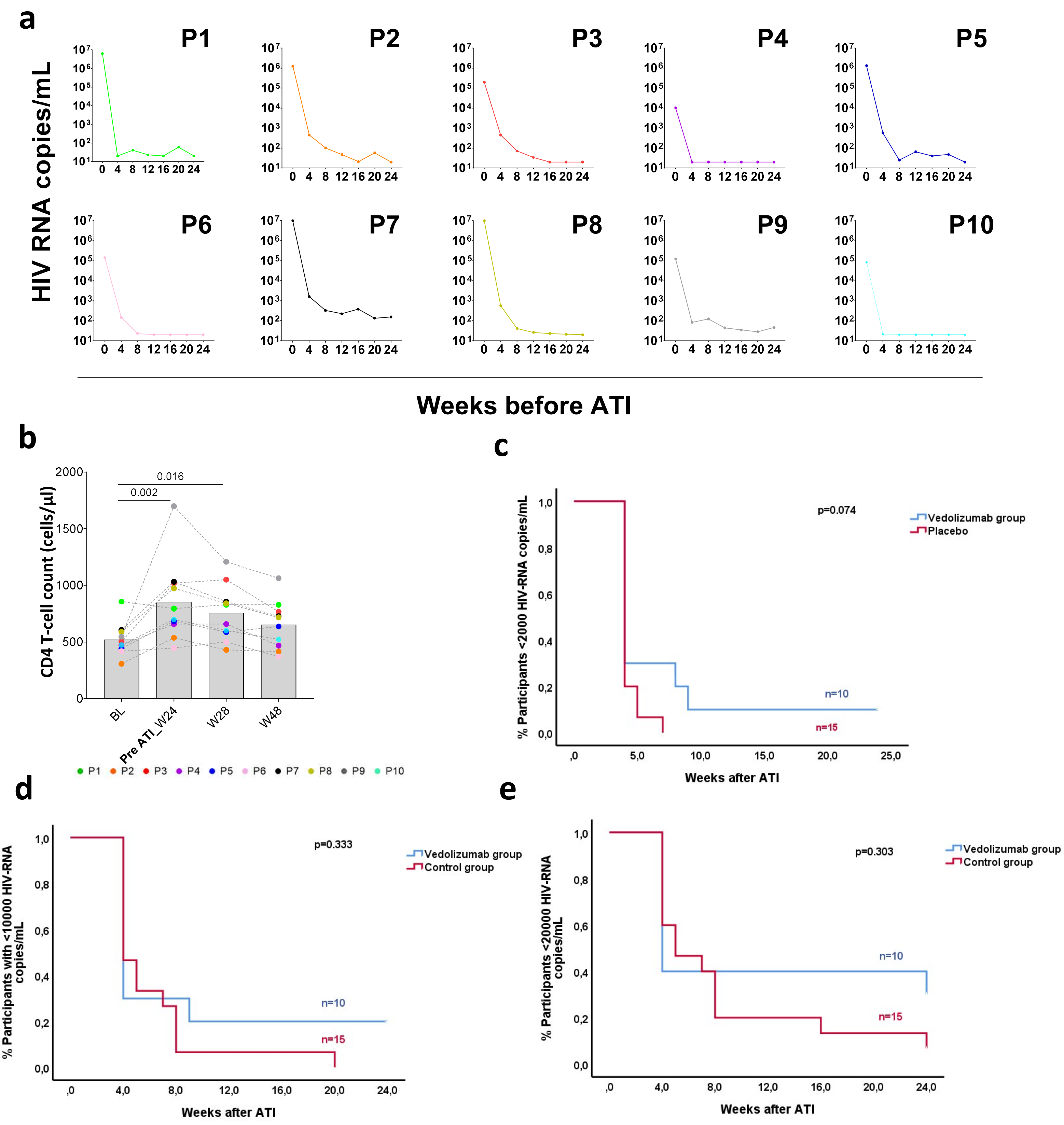

Supplementary Fig. 1. Plasma viral load kinetics before ATI and CD4+ T-cell levels along the follow-up. (a) Plasma viremia kinetics before ATI. **(b)** CD4+ T cell count along the follow-up. Friedman Test with Dunn's multiple comparisons test correction was used to assess differences along the follow-up. Abbreviations: BL, baseline; W, week; and ATI, analytic treatment interruption. **(c)** Kaplan-Meier analysis between vedolizumab and historical control group of the time to first VL>2000 HIV-RNA copies/ml, **(d)** >10000 HIV-RNA copies/ml and **(e)** >20000 HIV-RNA copies/ml.

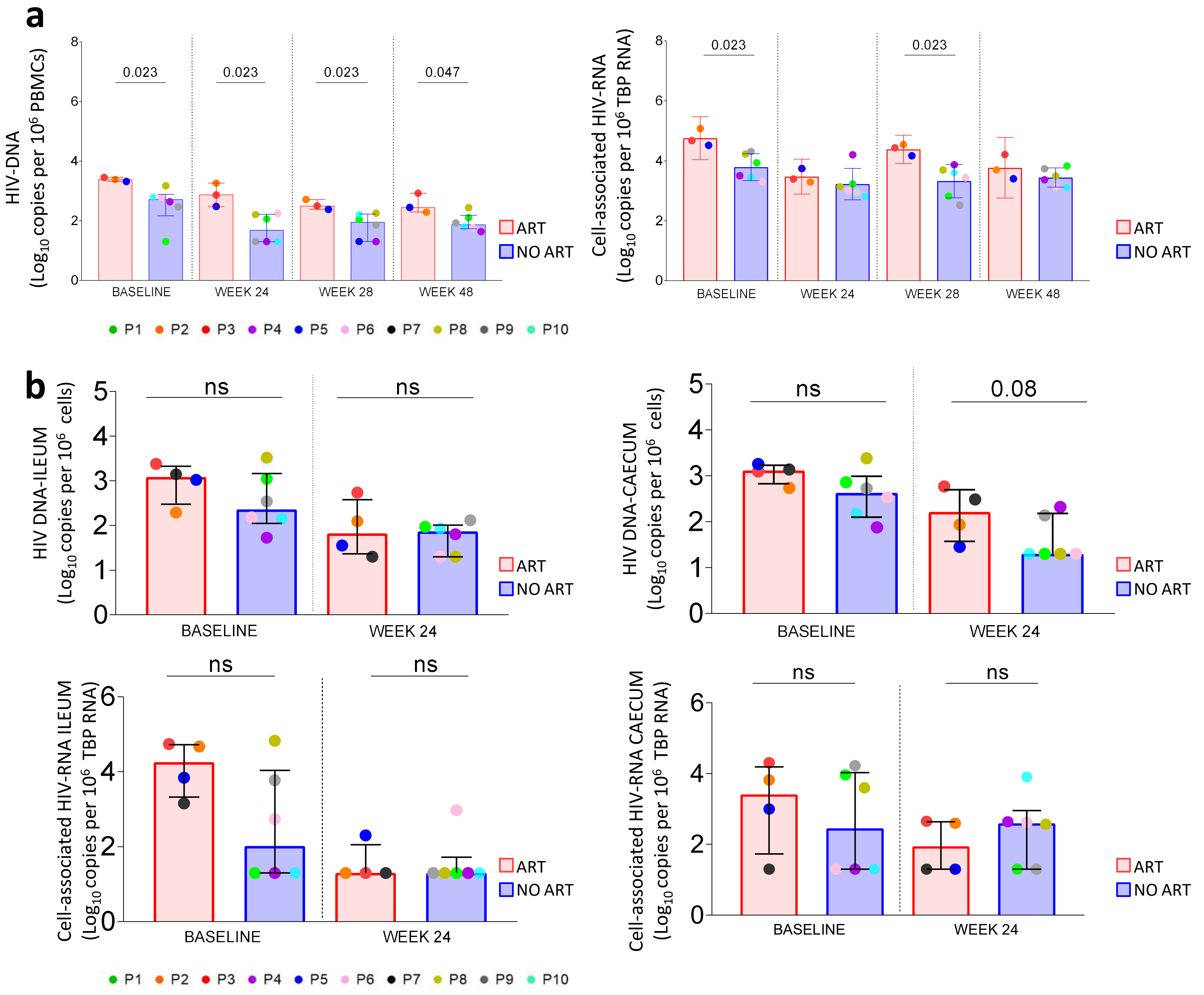

Supplementary Fig. 2. Dynamics of HIV reservoir in PBMCs and GITs. (a) Total HIV-DNA and cell-associated HIV-RNA levels in PBMCs along the follow-up. **(b)** HIV-DNA and cell-associated HIV-RNA levels in ileum (left panel) and caecum (right panel) before ATI. Mann-Whitney U test was used to assess differences. Red bars: Participants who restarted ART (P2, P3, P5 and P7) after ATI; Blue bars: Participants who did not restart ART (P1, P4, P6, P8, P9 and P10) after ATI. Abbreviations: BL, baseline; W, week and ART, antiretroviral therapy.

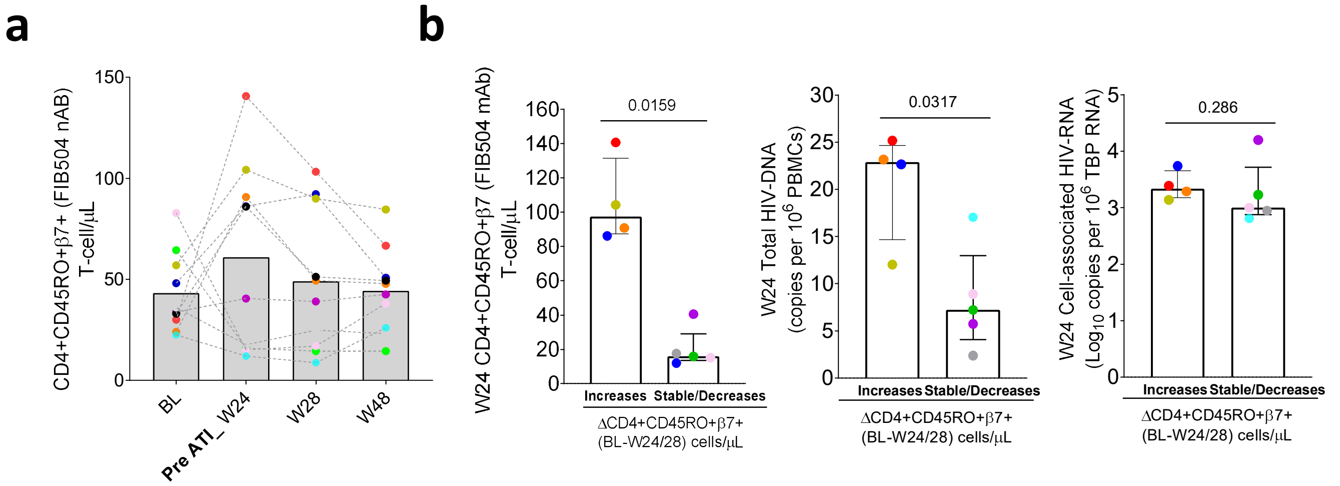

Supplementary Fig. 3. Analysis of the dynamic of CD4+CD45RO+β7+ T-cell counts and association with the size of the HIV-1 reservoir at week 24. (a) Dynamic of CD4+CD45RO+β7+ T-cell counts along the follow-up in PBMCs. **(b)** Correlation between dynamic patterns of peripheral CD4+CD45RO+β7+ T-cell counts at week 24/28 and memory CD4+ α4β7+ levels, total HIV-DNA, assayed by FLIP-seq, and HIV-RNA levels at week 24.

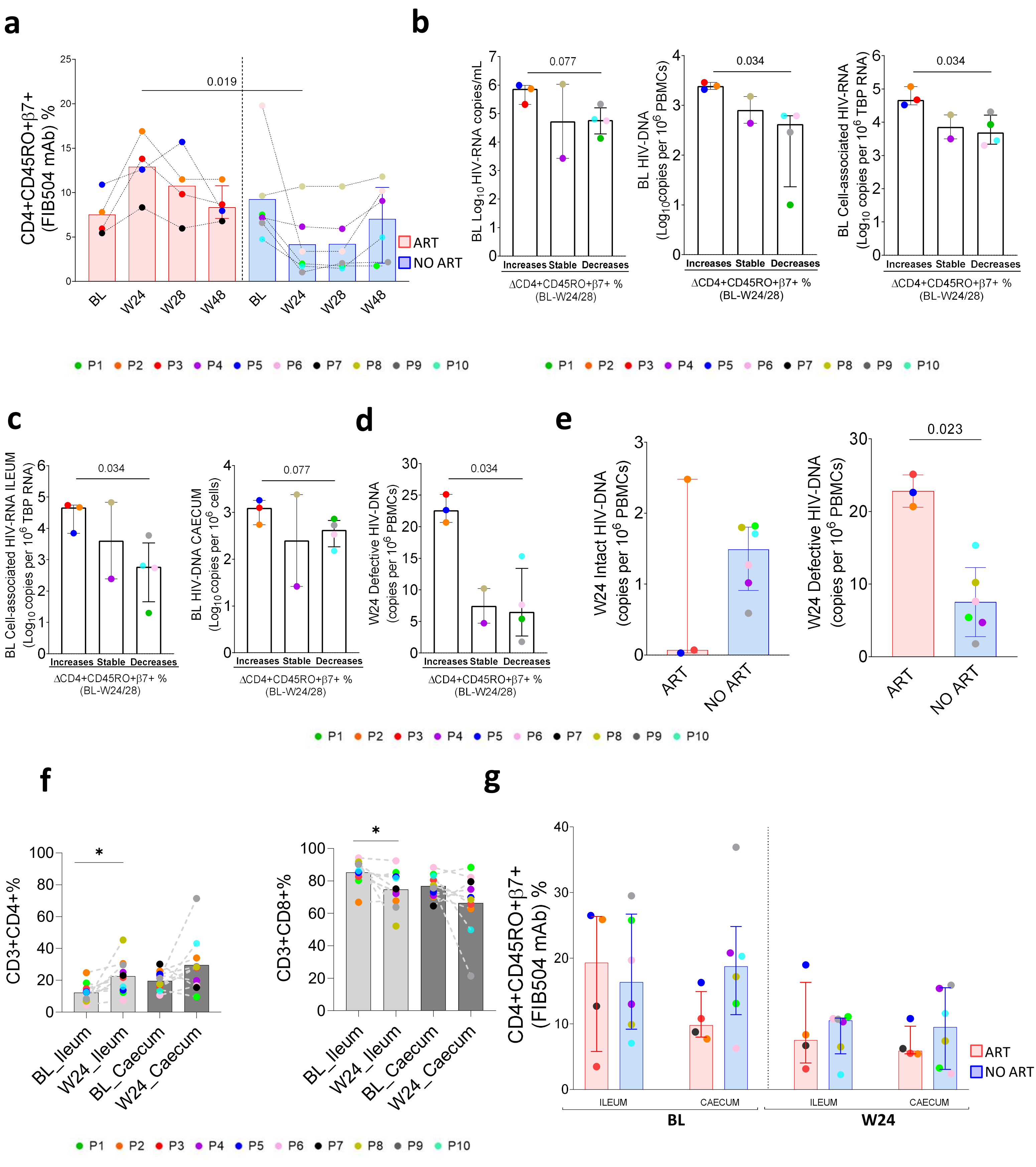

Supplementary Fig. 4. Analysis of the dynamic of β7 expression levels along the follow-up, associated with the size of the HIV-1 reservoir based on ART reintroduction. (a) Dynamic of α4β7 expression on CD4 T-cells along the follow-up in PBMCs based on ART reintroduction. **(b)** Correlation between dynamic patterns of peripheral CD4+ α4β7+ T-cell levels at week 24/28 and viral load, total HIV-DNA and HIV-RNA levels at baseline. **(c)** Correlation between dynamic patterns of peripheral CD4+ α4β7+ T-cell levels at week 24/28 and HIV-RNA levels in ileum and HIV-DNA levels in caecum. **(d)** Correlation between dynamic patterns of peripheral CD4+ α4β7+ T-cell levels at week 24/28 and defective HIV-DNA levels, assayed by FLIP-seq, at week 24. CD4+ α4β7+ levels were considered to decrease when there was >2,5 fold reduction at week 24/28 compared to BL. CD4+ α4β7+ levels were considered to increase when there was >1,3 fold change at week 24/28 compared to BL. **(e)** Intact and defective HIV-1-DNA, assayed by FLIP-seq, at week 24 based on ART reintroduction after ATI. **(f)** Dynamic of CD4+ and CD8+ T-cell levels in GIT at BL and week 24. **(g)** Dynamic of α4β7 expression on CD4 T-cells in ileum and caecum at BL and week 24 based on ART reintroduction after ATI. Wilcoxon and Mann-Whitney U test were used to assess differences between participants. Red bars: Participants who restarted ART (P2, P3, P5 and P7); Blue bars: Participants who did not restart ART (P1, P4, P6, P8, P9 and P10). Abbreviations: BL, baseline; W, week; and ART, antiretroviral therapy.

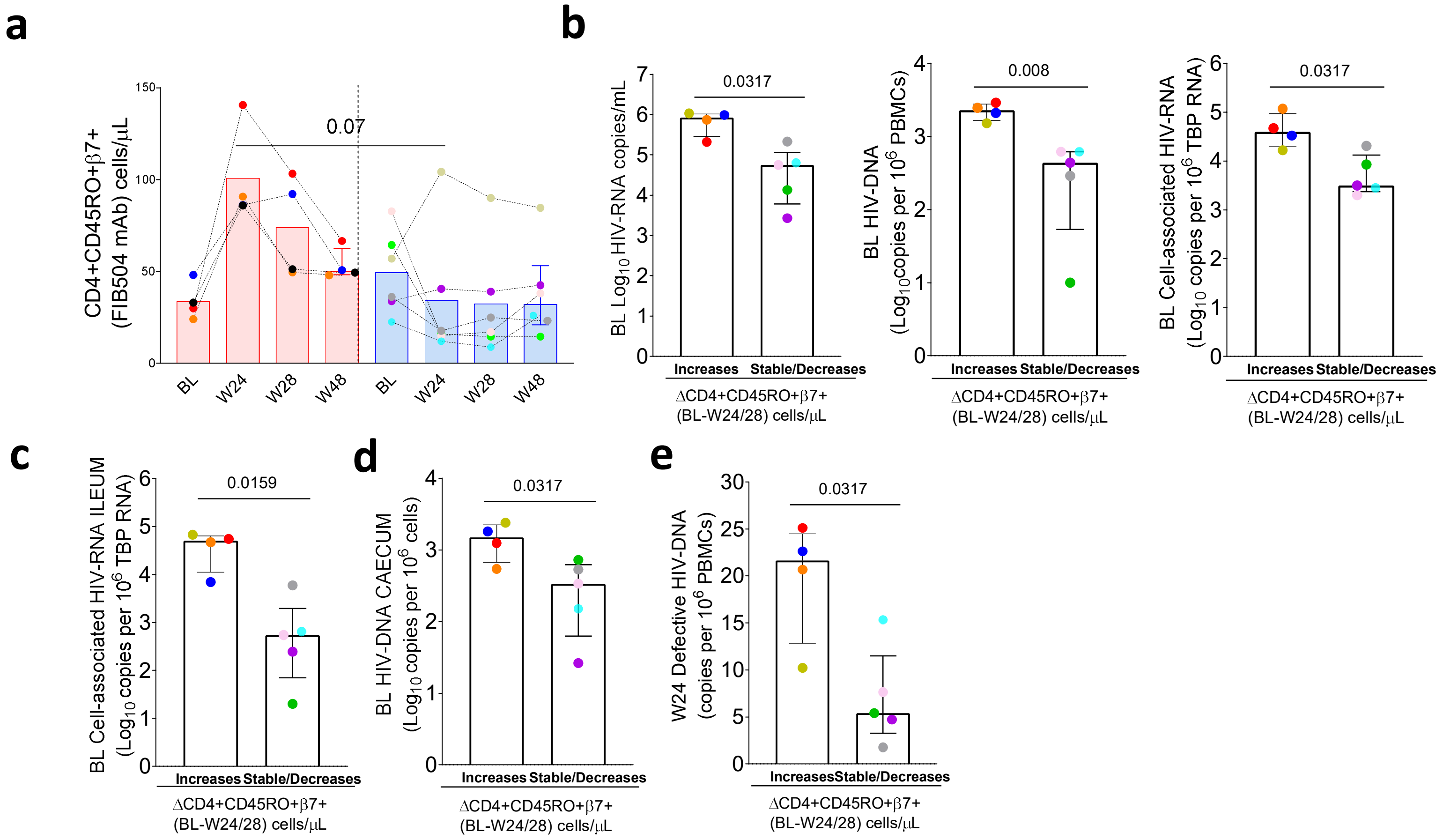

Supplementary Fig. 5. Analysis of the dynamic of CD4+CD45RO+β7+ T-cell counts along the follow-up, associated with the size of the HIV-1 reservoir based on ART reintroduction. (a) Dynamic of of CD4+CD45RO+β7+ T-cell counts along the follow-up in PBMCs based on ART reintroduction. **(b)** Correlation between dynamic patterns of peripheral of CD4+CD45RO+β7+ T-cell counts at week 24/28 and viral load, total HIV-DNA and HIV-RNA levels at baseline. **(c)** Correlation between dynamic patterns of peripheral of CD4+CD45RO+β7+ T-cell counts at week 24/28 and HIV-RNA levels in ileum and HIV-DNA levels in caecum. **(d)** Correlation between dynamic patterns of CD4+CD45RO+β7+ T-cell counts at week 24/28 and defective HIV-DNA levels, assayed by FLIP-seq, at week 24.

a

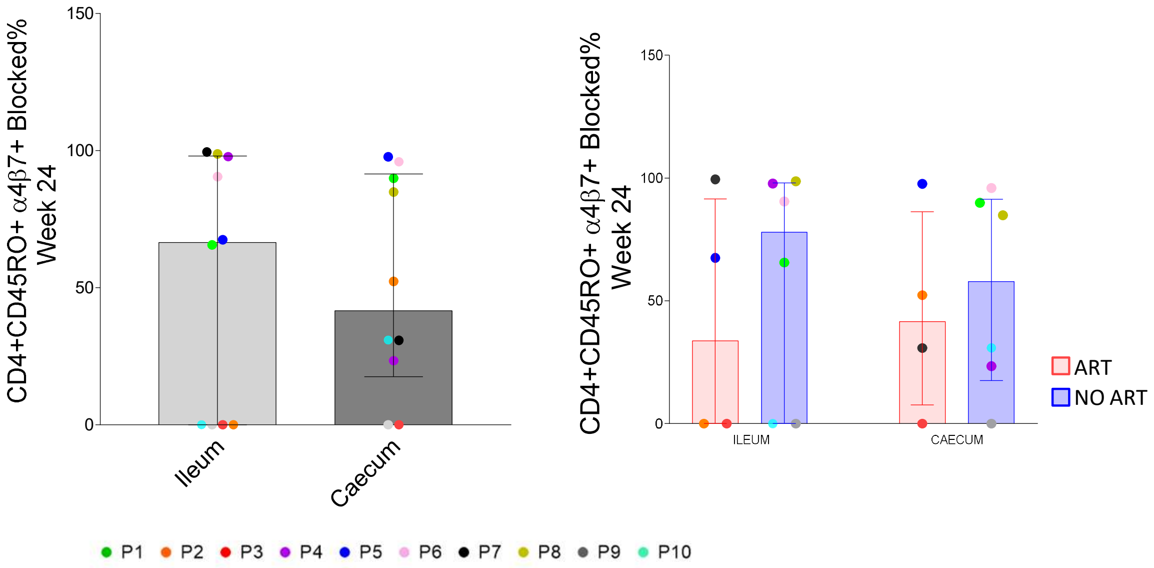

b

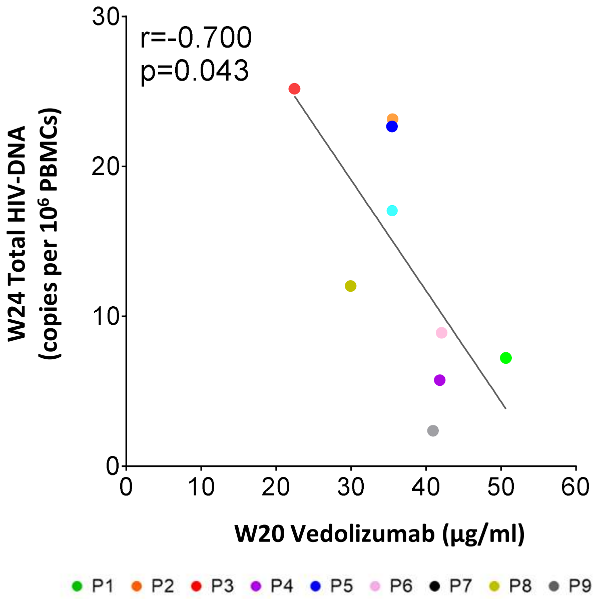

Supplementary Fig. 6. Levels of α4β7 blocking on CD4 + T cells in GIT based on ART reintroduction and association of HIV-DNA with vedolizumab levels. (a) α4β7 blocking levels in ileum and caecum based on ART reintroduction after ATI. **(b)** Correlation between serum concentration of vedolizumab and total HIV-1-DNA on PBMCs, assay by FLIP-seq, before ATI (week24). Red bars: Participants who restarted ART (P2, P3, P5 and P7); Blue bars: Participants who did not restart ART (P1, P4, P6, P8, P9 and P10). P values were computed using Mann-Whitney U and Spearman test.

a

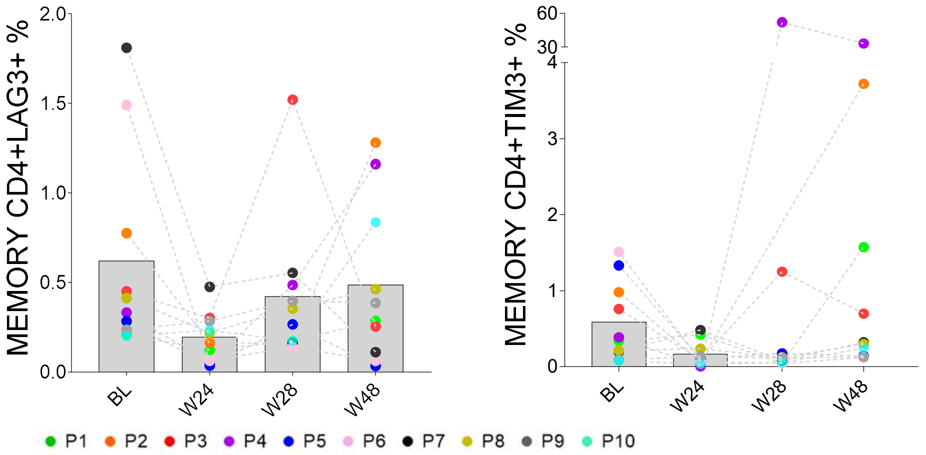

b

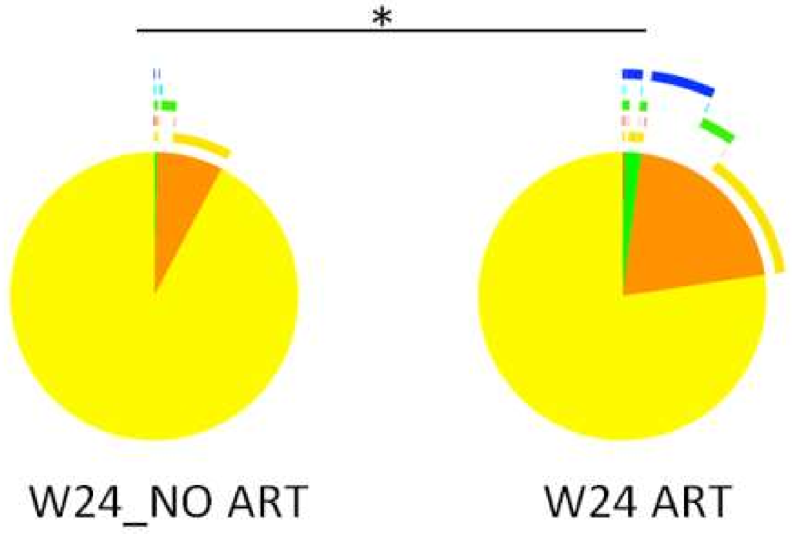

Pie Chart Arc Legend											
β7Integrin		LAG3		PD1		TIGIT		TIM3			
**Pie Chart Portion Legend**											
5 Markers		4 Markers		3 Markers		2 Markers		1 Markers		0 Markers	

c

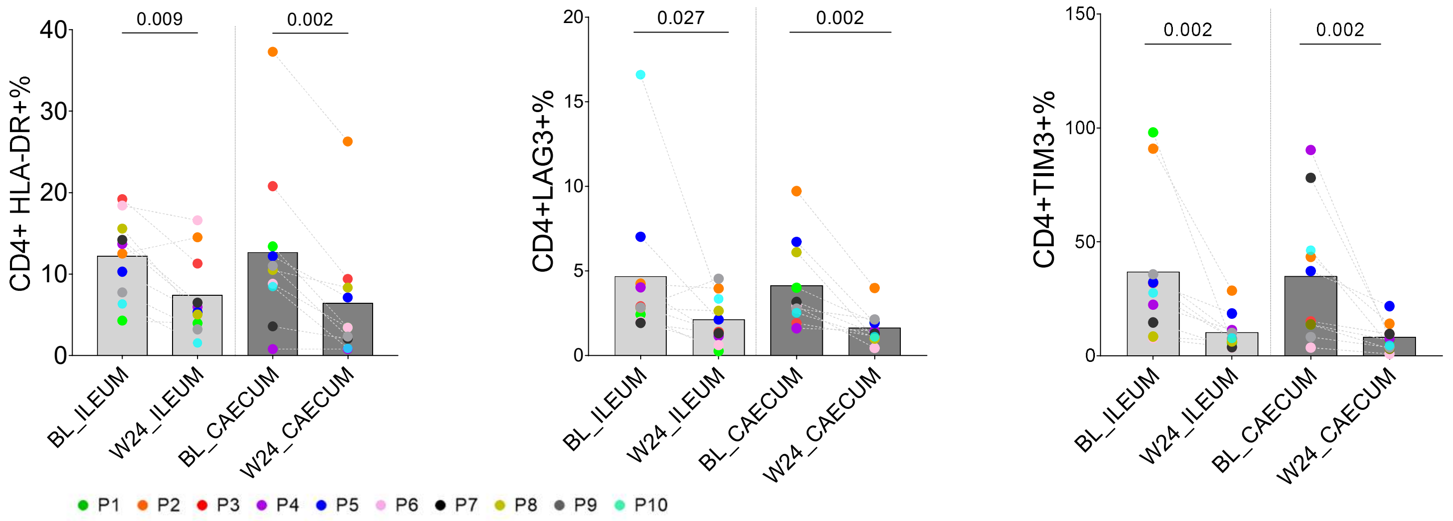

Supplementary Fig. 7. Immune checkpoint molecules and activation markers levels on PBMCs and GIT. (a) TIM3+ and LAG3+ memory CD4+ T cell levels along the follow up on PBMCs. **(b)** Immune check point molecules (LAG3, PD1, TIGIT and TIM3) coexpression on memory CD4+ T-cells. Section in the pie charts represent the proportion of simultaneous expression of the number of markers. The arcs represent the different markers in each section. **(c)** HLA-DR+, LAG3+ and TIM3+ CD4+ T-cells in GIT along the follow up. *p* values were computed using Friedman Test with Dunn's multiple comparisons test correction, Wilcoxon and Mann-Whitney U test, and with permutation test for pie charts.

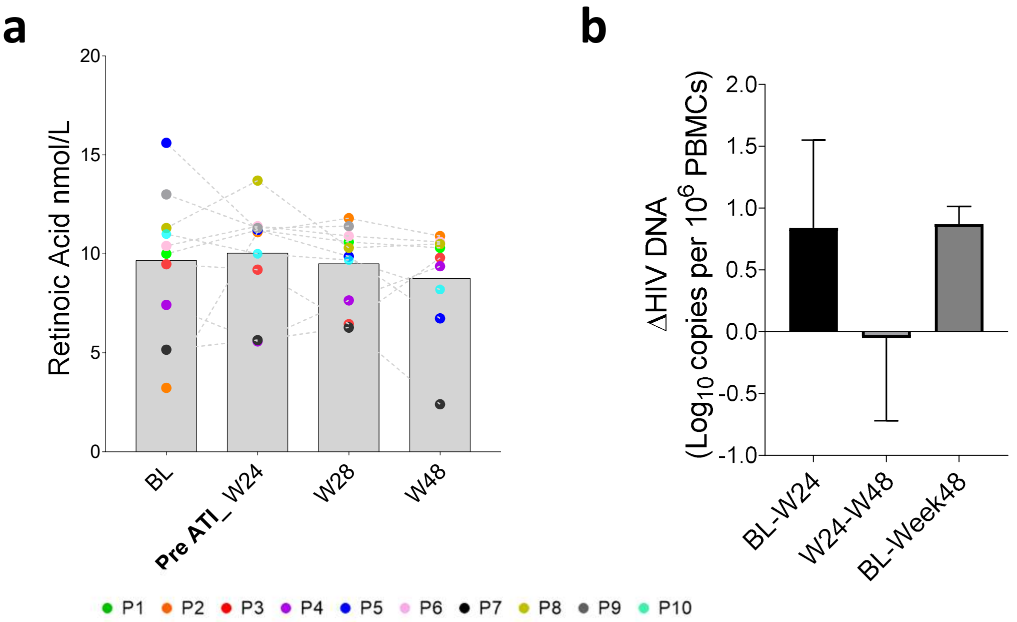

Supplementary Fig. 8. Retinoic acid plasma levels and changes in HIV-DNA in PBMCs. (a) Retinoic acid plasma levels along the follow up. **(b)** Changes in HIV-DNA at different follow up time points. Friedman Test with Dunn's multiple comparisons test correction to assess differences along the follow-up. Abbreviations: BL, baseline; W, week; and ATI, analytic treatment interruption.

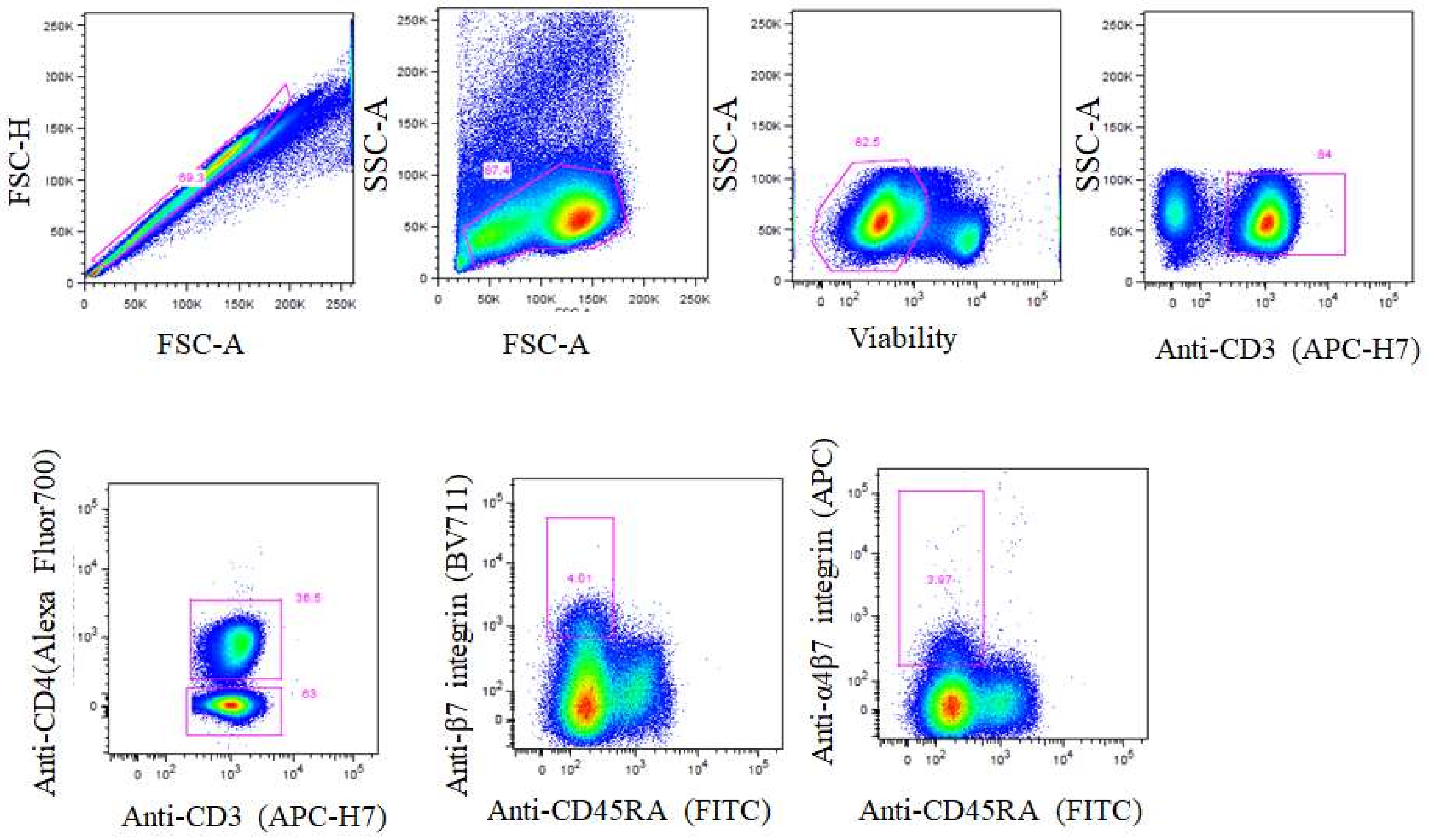

Supplementary Fig. 9. Quantification of CD4+ α4β7+ cells. Gating strategy.
